# Drug metabolism and pharmacokinetics of praziquantel: A review of variable drug exposure during schistosomiasis treatment in human hosts and experimental models

**DOI:** 10.1371/journal.pntd.0008649

**Published:** 2020-09-25

**Authors:** Grace Zdesenko, Francisca Mutapi

**Affiliations:** 1 Institute of Immunology & Infection Research, University of Edinburgh, Ashworth Laboratories, King’s Buildings, Edinburgh, United Kingdom; 2 NIHR Global Health Research Unit Tackling Infections to Benefit Africa (TIBA), University of Edinburgh, Ashworth Laboratories, King’s Buildings, Edinburgh, United Kingdom; University of Calgary, CANADA

## Abstract

Schistosomiasis control is heavily reliant on the drug praziquantel (PZQ), which is used as preventive chemotherapy as part of national helminth control strategies. Given the heavy reliance on PZQ for mass drug administration, there has been considerable research on the potential of parasites developing resistance to the drug, resulting in decreased drug efficacy. However, there have been comparatively fewer studies of other factors that can potentially alter PZQ efficacy. Here, we investigate whether host PZQ metabolism contributes towards variable cure rates. We evaluate factors that can influence the metabolism of PZQ and the resultant effect on the efficacy of PZQ treatment to determine factors that potentially influence an individual’s response to the drug. The literature search was directed at published studies from three online databases: Web of Science, PubMed, and EMBASE. The search terms for the review comprised of ([praziquantel OR PZQ] AND [schistosom* OR bilharzia] AND [pharmaco*]) and included studies evaluating PZQ metabolism. Publications were categorised into pharmacokinetics, drug–drug interactions, pharmacogenetics, and metabolite analysis. Forty publications describing human and experimental studies fitted the inclusion criteria and were subjected to data extraction and analysis. The analyses showed that variable exposure to PZQ was associated with alterations in the liver’s capacity to metabolise PZQ and observed drug–drug interactions. Other factors influencing the efficacy of PZQ were brand, formulation, and co-administered food. Although some work has been performed on metabolite identification, there was minimal information on PZQ’s metabolic pathway, and no pharmacogenetics studies were identified. The study indicated that in both human and experimental studies alterations in the liver’s capacity to metabolise PZQ as well as drug–drug interactions affected systemic levels of PZQ that could result in variable cure rates. The study confirmed previous findings of higher antischistosomal activity of (R)-PZQ enantiomer when administered alone compared to the racemate at the same dose as well as improved efficacy when the drug is administered with food. The study also highlighted the need for more comprehensive studies of the PZQ metabolic pathway and PZQ pharmacogenetic studies in humans.

## Author summary

Schistosomiasis is a neglected tropical disease caused by parasitic worms, and its control is heavily reliant on the drug praziquantel (PZQ). Reports of individuals not being cured of schistosomiasis after a treatment have raised concerns about drug efficacy and the potential for the development of drug resistance. Many factors can be responsible for reduced efficacy including host, parasite, and drug–related factors. We investigated the potential effect of varying systemic levels of PZQ on cure rates. We reviewed studies on PZQ metabolism identifying factors that may influence PZQ systemic levels, including the drug administered, host liver health, and genetic polymorphisms of liver enzymes involved in PZQ metabolism. The results indicated that varying levels of PZQ were due to the drug formulation and differences in the liver’s capacity to metabolise PZQ as well as drug–drug interactions. The study also highlighted the need for further studies of the effect of human genetics on PZQ’s metabolic pathway, and the PZQ metabolites produced.

## Introduction

**Fig 1:**
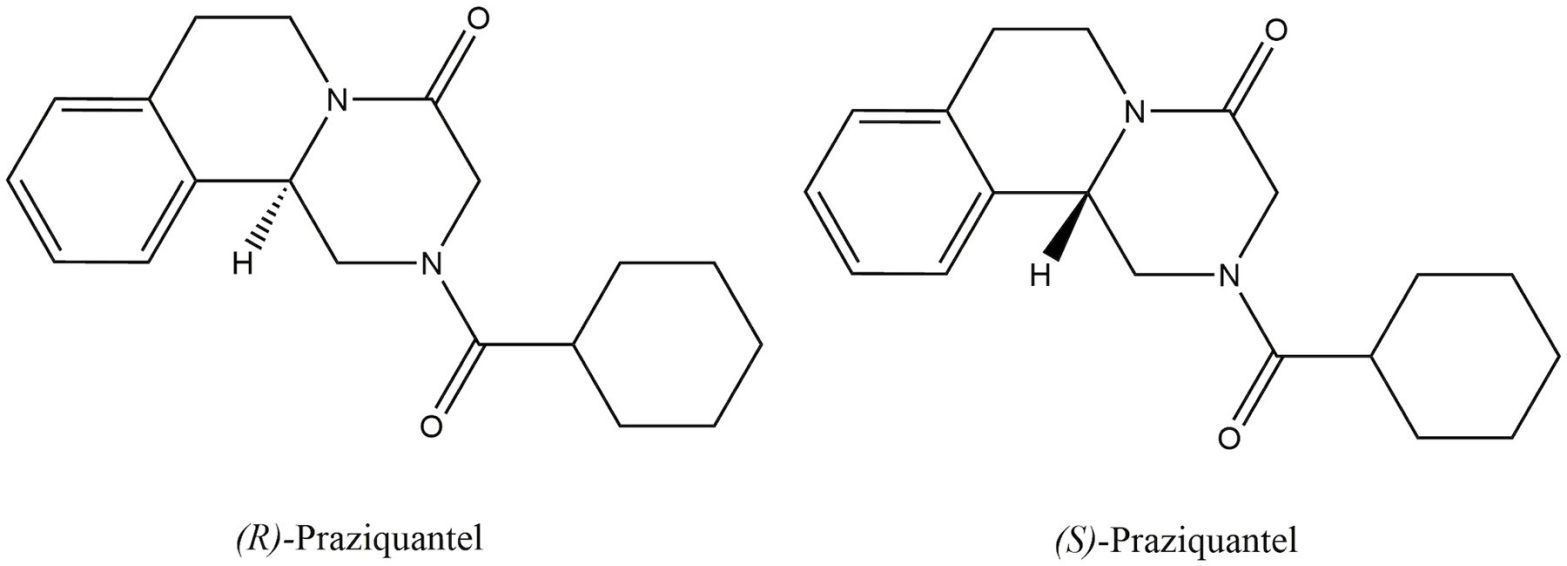
The molecular structure of the two PZQ enantiomers. The (R)-praziquantel has the hydrogen atom (H) pointing down from the chiral center. The (S)-praziquantel has the hydrogen atom (H) pointing up from the chiral center. PZQ, praziquantel.

Schistosomiasis is a prominent public health problem [1], with the majority of affected people residing in Africa [2]. Praziquantel (PZQ) is the drug of choice to treat schistosomiasis and is widely used in preventive chemotherapy (PCT) programs (as defined by WHO) [3] across Africa to treat intestinal and urogenital schistosomiasis infections caused by *Schistosoma mansoni* and *S*. *haematobium* parasites, respectively [4]. Mass drug administration (MDA) of PZQ in PCT to treat schistosome infection and reduce associated morbidity has been a success, with an estimated 235 million people treated with PZQ in 2018 alone [5]. PZQ itself is a racemic drug, with the standard dose consisting of a 1:1 mixture of two enantiomers (see Fig 1).

Only the (R) enantiomer (also known as Levo-PZQ, L-PZQ, or (-)-PZQ) of PZQ has antischistosomal activity; in contrast, the (S) enantiomer (also known as Dextro-PZQ, D-PZQ or (+)-PZQ) does not have antischistosomal action but contributes to some of PZQ’s known side effects [6]. PZQ is well-tolerated and effective in patients of all ages with different clinical forms of schistosomiasis [7] and has been used to treat schistosomiasis since the 1980s [8]. The precise mechanism of PZQ’s antiparasitic action remains poorly described. Studies suggest that its action arises from the (R)-PZQ enantiomer disrupting the schistosome calcium ion homeostasis causing uncontrolled muscle contraction and death in adult worms [9, 10]. PZQ targets only adult schistosomes; therefore, the drug is not effective against the larval stages infections [11].

The efficacy of PZQ treatment is determined by the cure rate (CR), which compares the number of egg-positive individuals pre-PZQ treatment who become negative for schistosomiasis post-PZQ treatment as well as by the egg reduction rate (ERR), determined by the reduction in mean number of eggs excreted in urine or stool (depending on the schistosome species) from pre-PZQ to post-PZQ treatment [12, 13]. Low PZQ cure rates have been reported by some studies, with many attributing this to a high pretreatment parasite burden [14–17]. However, variable efficacy of PZQ has also been observed in other studies, even when accounting for the level of pretreatment infection [18, 19], suggesting that other factors must be influencing the drug’s efficacy. For example, low cure rates and the reduced efficacy of PZQ treatment have been attributed to patients harbouring schistosomes in different developmental stages and the decreased sensitivity of schistosomes to PZQ treatment [20–23]. Investigations into cases of laboratory and field PZQ resistance indicated that reduced efficacy of drug treatment due to decreased sensitivity and resistance was rare [24, 25]; hence, parasite sensitivity cannot account for all incidences of treatment failure. So, why are these low cure rates occurring? This study investigates whether the level of PZQ in systemic circulation is contributing towards low cure rates, as systemic PZQ may not be exceeding the lethal schistosome concentration. In this study, we will focus on three factors that can influence the amount of PZQ in systemic circulation, and, thus, affect the efficacy of PZQ treatment: PZQ pharmacokinetics (PK), pharmacogenetics, and drug–drug interactions.

The term “PK” describes movement of a drug, encompassing the absorption, distribution, metabolism, and elimination (ADME) parameters of that drug [26]. Metabolism describes the mechanism of breaking down the drug compound and is commonly analysed via biotransformations of the xenobiotic by drug-metabolising enzymes [27]. The Cytochrome P450 (CYP) enzymes mediate the metabolism of PZQ, specifically the following enzymes: CYP1A2, CYP2C9, CYP2C19, CYP2D6, CYP3A4, and CYP3A5 [28]. The term pharmacogenetics refers to the variability in an individual’s response to a drug due to genetic variations. Genetic polymorphisms in CYPs have linked interindividual variation to metabolism in numerous drug efficacy and toxicity studies [29]. These variations are primarily due to single nucleotide polymorphisms (SNPs) of the CYPs, and these can lead to an increased or decreased pharmacokinetic effect [30]. The distribution of CYP alleles differs substantially between populations, emphasising the need for optimising drugs for the population in which the drug will be used, e.g. the efficacy of drugs tested in Europe may not have the same efficacy in African populations [31]. Furthermore, analysing pharmacogenetic differences in the metabolic (drug metabolism) products can provide additional information on a patient’s drug response [32].

The CYPs are also an important site for drug–drug interactions (DDIs) [33]. PZQ is metabolized by multiple CYPs, and so DDIs within these CYP pathways could result in the formation and accumulation of metabolic by-products or a reduction of the drug’s therapeutic effect [34]. The bioavailability of a drug can be altered by DDIs via competition for protein-binding sites on the CYPs, affecting the overall efficacy of a drug. DDIs can induce the CYPs (increased metabolism), increasing the activity of the CYP enzymes and decreasing the overall bioavailability of the active drug [35]. Vice versa, if a coadministered drug inhibits the PZQ’s enzymatic binding sites, it can no longer be metabolised and eliminated via this pathway (decreased metabolism), and drug accumulation could result in toxic levels in the body [36]. Evaluating DDIs is important when developing and using any drug treatment, especially when regarding comorbidity, as patients may be on multiple medications [37]. Overall, evaluating whether pharmacogenetics factors and DDIs influence a CYP’s ability to metabolise PZQ will indicate the role of altered metabolism in variable schistosomiasis CR.

## Methods

### Literature search strategy

The search strategy was guided by the Preferred Reporting Items for Systematic Reviews and Meta-Analysis (PRISMA) guidelines [38], and the studies included in this review were published prior to May 12, 2019. The search used three online databases: Web of Science, PubMed, and EMBASE. The search terms for the review comprised of ([praziquantel OR PZQ] AND [schistosom* OR bilharzia] AND [pharmaco*]) and included animal and experimental models that evaluated PZQ metabolism. As the main burden of schistosomiasis in sub-Saharan Africa is attributed to two species of schistosomes [4], only studies on *S*. *haematobium* and *S*. *mansoni* were included in this review. The citations were compiled in EndNote X8 and duplications were identified and removed.

### Literature screening and inclusion and exclusion criteria

The titles and abstracts of the Endnote Library were screened, and any that met the inclusion criteria were put aside for a review of the full text. If the abstract was not available or it was unclear whether the study fully met the inclusion criteria, it was selected for full evaluation. After a full evaluation of each text, the articles included in this review were required to fit into one of the following topics: PK, pharmacogenetics, drug–drug interactions, or metabolites analysis. Selection criteria within each category were based on whether (1) the article was available as full journal text and in English, (2) the units of the numerical pharmacokinetic parameters were clearly defined, (3) the article included human and animal *in vivo*/*in vitro* models for human extrapolation, and (4) models were either a healthy control or infected with *S*. *mansoni* or *S*. *haematobium*. If a paper could not be located through an online repository, it was requested from the British Library. Any papers that could not be obtained from these sources were excluded.

### Data extraction and data analysis

Pharmacokinetic data, including the dose of PZQ and any “Drug B” measured (racemic or enantiomeric PZQ), the model, *Schistosoma* infection status, the number of subjects (N), the numerical pharmacokinetic parameter and its units, and any external test conditions, such as fasting or fed states, were documented in an Excel spreadsheet. To extract comparable data from each study, it was concluded that the *in vivo* pharmacokinetic parameters to quantify drug-concentration-time relationships would be as follows: area under the plasma-concentration curve (AUC), peak plasma concentration (C_max_), time to reach peak plasma concentration following drug administration (T_max_), and drug-elimination half-life (t_1/2_) [39]. Data points were extracted, grouped together by parameter, and converted into standard SI units. If no units were quoted, the data point was excluded from the data tables. Where the published data was presented in a graph, Data Thief III software was used to extract the numerical values. The data points were analysed to detect trends affecting PZQ metabolism between studies, creating new combined graphical results. These graphs were then analysed in the results to assess whether the combined data reflected the hypothesis of this review. Only limited statistical analysis was possible due to sparsity of data and heterogeneity of populations used.

To evaluate the efficacy of PZQ without pharmacokinetic values, the worm burden reduction (WBR) in the case of experimental models, ERR, and CR were also collected. For pharmacogenetic factors, the population ethnicity of each human study was recorded when provided in the source publication as genetic factors can determine response to a drug [40]. By linking the individuals in the studies by ethnicity, it implies a common genetic ancestral background that could influence a therapeutic response to a drug [41]. For the papers that depicted PZQ metabolite analysis, the structural identity of each metabolite was created using ChemDraw Prime v16.0, and any information about its metabolic pathway was reported.

## Results

The search yielded a combined total of 873 studies and, after the removal of duplicates, 425 titles and abstracts were screened, resulting in 95 publications for a full-text review. There were 55 excluded publications, leaving 40 publications for data extraction and analysis (S1 Table). The results of this systematic search are displayed in a flow chart (S1 Fig).

### PK and PZQ efficacy

In the PK category of this review, data points extracted from multiple papers describing healthy and infected human and animal models provided an overview of the published PK and efficacy of PZQ treatment. The animal models obtained from the search strategy were exclusively rodents, and included mice, rats, and hamsters. The human studies included adults (16 years or older), school-aged children (SAC) (6 to 15 years), and preschool-aged children (PSAC) (5 years or younger). An evaluation of these as separate study groups was made based on the model, either rodent or human, and parameters measured.

### Experimental studies: PK and PZQ efficacy

The relative exposure of PZQ in the reviewed rodent models was compared using t_1/2_, T_max_, C_max_, and AUC values (refer to S2 Table). The effects of different PZQ doses, brands, and infection status on the exposure parameters (C_max_ and AUC) of PZQ in various rodent models were described in six studies [42–47].

#### Hepatic CYP metabolism

On average, based on the PK data extracted from these papers, the general trend of the compiled exposure parameters was that with increasing PZQ dose, the C_max_ and AUC also increased. Upon closer inspection, there were some deviations from this trend with a common factor; it appeared that the healthy rodent models tended to have a lower exposure to PZQ than the infected rodents. A study by Botros and colleagues [42] in healthy and *S*. *mansoni*–infected mice showed that in the infected mice there was a significant difference in maximum concentration of PZQ in systemic circulation, approximately triple that found in healthy mice. The increased exposure was attributed to the reduced liver capacity of infected rodents, resulting in decreased metabolism of parent PZQ and, therefore, longer exposure in the circulation. Botros and colleagues [43] and Kokwaro and colleagues [44] conducted further analysis of the overall decreased hepatic CYP activity and the resultant alteration of PZQ’s PK parameters due to schistosomiasis, once again with significant differences between *S*. *mansoni*–infected and uninfected mice detected. The relative t_1/2_, T_max_, C_max_, and AUC values were increased proportionally to the degree of decreased CYP activity, an influential factor if this effect is also observed in humans. In fact, due to decreased hepatic CYP activity, severe side effects were observed by Gotardo and colleagues [45] from toxicity caused by the higher C_max_ and AUC values in infected mice treated with 400 mg/kg PZQ.

#### PZQ brands

Additional factors which may have contributed to variable PZQ exposure were investigated by comparing the parasitic efficacy of the different brands of PZQ available. To compare efficacy, two parameters were measured: (1) WBR, as the World Health Organization describes a reduction in adult worm counts as a measure of the efficacy of an anthelmintic in experimental models [48], and (2) the percentage inhibition of hepatic CYP450 activities as a result of *S*. *mansoni* infection, observing the extent of CYP450 inhibition when compared to healthy mice. The results in S3 Table are adapted from Botros and colleagues [42], with the highest WBR and lowest CYP450 inhibition was achieved with pure PZQ, with Distocide and Biltricide showing comparable efficacy. Interestingly, Bilharzid, brand T3A, and Epiquantel had a significantly lower WBR than the other brands analysed, yet had significantly higher CYP450 inhibition. In comparison to pure PZQ, Distocide and Biltricide, the Bilharzid, brand T3A, and Epiquantel were not aiding the recovery of the CYP450 activity from schistosomiasis infection as well as the other brands examined.

#### PZQ formulations

**Fig 2:**
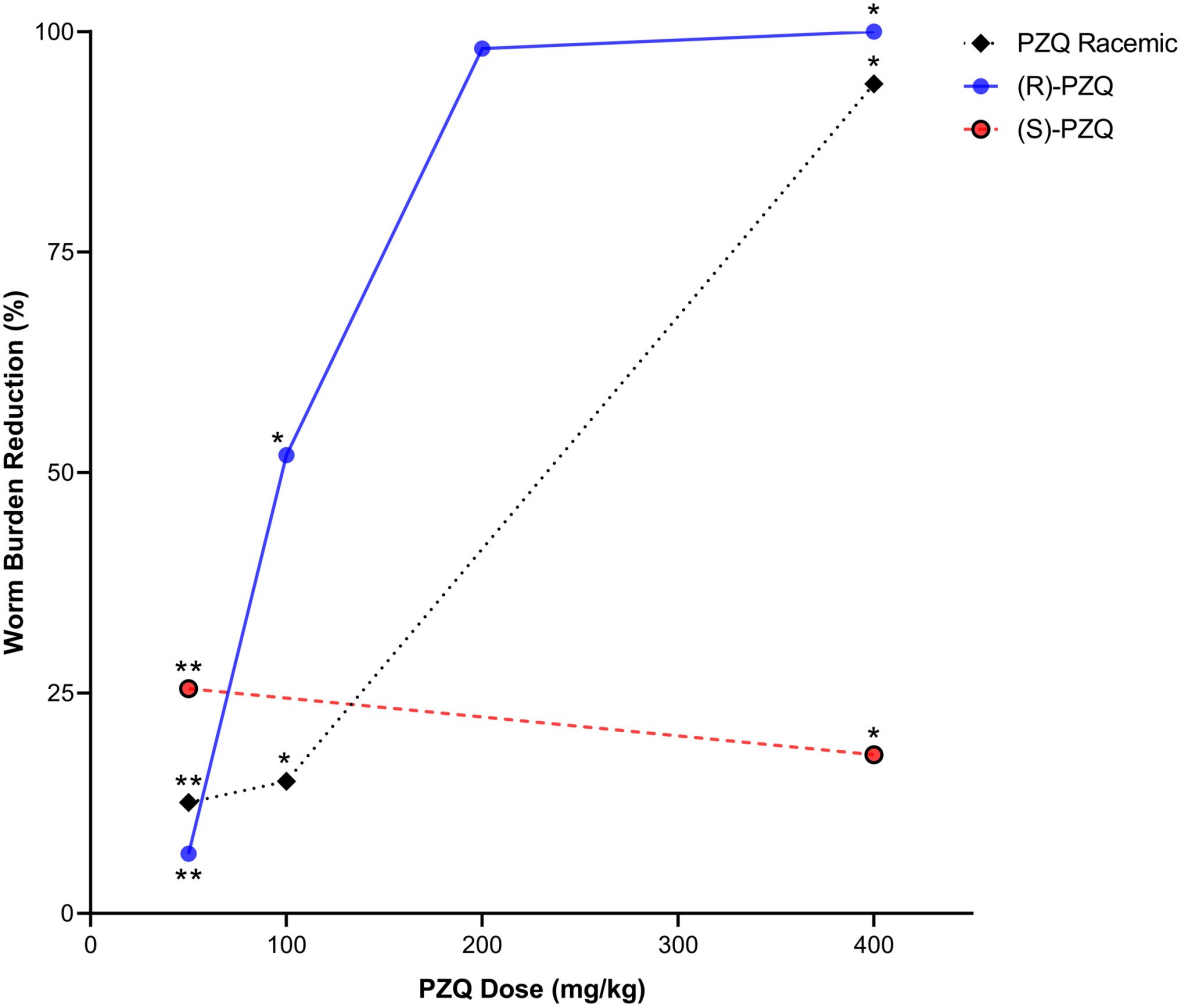
Comparison of the percentage WBR in an infected mouse model when dosing racemic PZQ or a single enantiomer. The data for this graph were extracted from two papers in this review and evaluated to assess the WBR-dose response [49, 50]. The relationship between WBR and drug formulation was found to be significantly different (*P < 0.01, **P < 0.001) for each PZQ dose based on the results of the independent samples Kruskal-Wallis Test. PZQ, praziquantel; WBR, worm burden reduction.

Further studies focused on the PZQ compound itself as a topic of discussion, with Zhang and colleagues [46] and Meister and colleagues [49] showing the pharmacological differences between the (R) and (S)-PZQ enantiomers, confirming the antischistosomal activity of (R)-PZQ. A comparison of the activity of racemic PZQ (rac-PZQ) compared to each PZQ enantiomer in mouse models can be seen in Fig 2 (data in S4 Table).

This is consistent with (R)-PZQ being the pharmacologically active enantiomer, with a significantly higher WBR than the (S)-PZQ and a higher WBR than the current standard rac-PZQ treatment at the same dose [49, 50]. One contradictory piece of data extracted during this review was from Tanaka and colleagues [50], in which the (S)-PZQ had a higher WBR than rac-PZQ and (R)-PZQ at 50 mg/kg of PZQ, yet this could be due to interanimal variability as the study was only conducted in seven mice. Overall, this discrepancy in the activity of the enantiomers was only observed in one paper and was not seen in any of the human studies.

A PZQ formulation (Polymorph B) was tested in mice by Lombardo and colleagues [47] as a new treatment option in comparison to the current PZQ tablet. Polymorph B aimed to increase efficacy and improve the PK parameters of the current PZQ formulation, which has low bioavailability and low water solubility, by enhancing PZQ’s solubility and dissolution. The crystalline formulation created from grinding rac-PZQ showed improved physical properties, particularly increasing PZQ chemical stability and doubling water solubility. Yet, the PK parameters of both enantiomers of Polymorph B had a lower exposure profile compared to a reference PZQ, indicating lower bioavailability of the new drug formulation compared to the current standard PZQ. This was most prominently visualised in the AUC value, which was approximately 40% lower for Polymorph B than reference PZQ.

### Human studies: PK and PZQ efficacy

As with the rodent models, the relative exposure of PZQ was compared using t_1/2_, T_max_, C_max_, and AUC values, with 13 papers containing human PK data sets [51–63].

#### PZQ formulations

**Fig 3:**
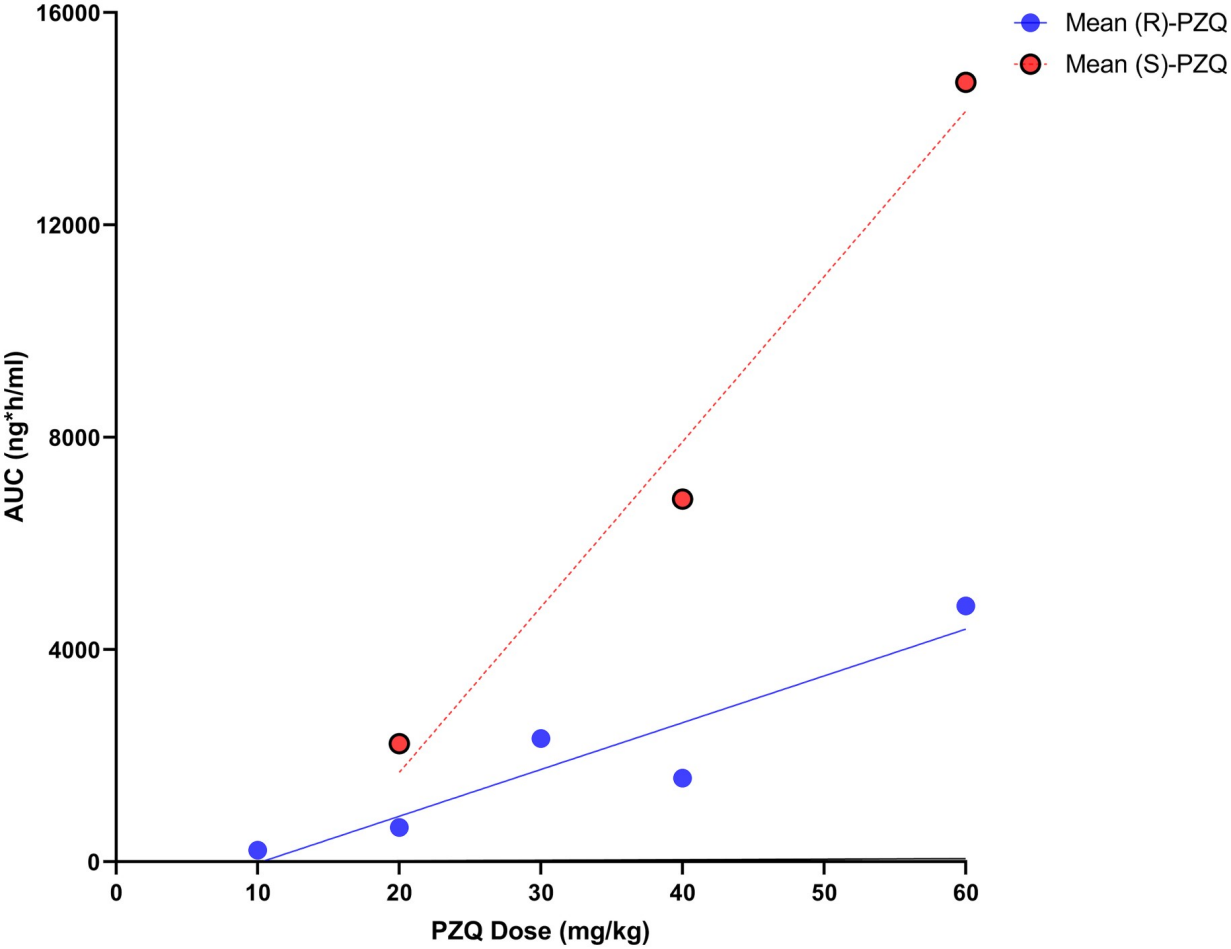
The mean AUC values extracted from the included studies against the PZQ dose of each of the PZQ enantiomers in HNV. The PZQ enantiomers were measured after a racemic PZQ dose or the (R)-PZQ dosed alone. These data were extracted from multiple papers in this review, separated by PZQ enantiomer, and the AUC was averaged for each dose and plotted on a graph for analysis [51–54]. Using a linear regression model, the difference in AUC between (R)-PZQ and (S)-PZQ in humans was found to be statistically significant (*P* < 0.05). AUC, area under the curve; HNV, healthy normal volunteers; PZQ, praziquantel.

As seen in the rodent models, data in human studies also showed a significant difference in antischistosomal activity between the two PZQ enantiomers. Four papers [51–54] (S5 Table) measured the PK of each PZQ enantiomer after a racemic dose in humans. The results were compiled and, as Fig 3 depicts, there were clear differences in enantiomer exposure between the biologically active (R)-PZQ and the (S)-PZQ.

Despite (R)-PZQ being the desired circulating pharmacoactive substance, the results extracted during this review in healthy normal volunteers (HNV) showed that the exposure parameters of the (R)-PZQ enantiomer were significantly lower than the (S)-PZQ. Therefore, the C_max_ and the AUC of the active enantiomer are lower than the inactive enantiomer at the same dose (Fig 3), so, when PZQ is dosed as a racemic tablet, there are significantly lower proportions of the desired enantiomer entering the circulation. This is the opposite of the desired action and demonstrates that a dose with a rac-PZQ tablet is not reflective of the dose of active (R)-PZQ.

Two studies in this review evaluated the PK of a small orally dispersible tablet (ODT) formulation that would allow for fast dispersion and an acceptable taste [51, 52]. The relative bioavailability of a rac-PZQ ODT formulation was compared with the single active enantiomer (R)-PZQ ODT ((R)-ODT), to determine if there were pharmacokinetic differences between ODTs and the current rac-PZQ tablet. When dosed at 40 mg/kg, the rac-PZQ reference is 50:50 of (R)-PZQ to (S)-PZQ; therefore, it was expected that when the (R)-ODT was dosed at 20 mg/kg, it would be approximately equal to that of the 40 mg/kg of rac-PZQ or the PZQ reference. However, this did not appear to be the case as the 20 mg/kg dose of (R)-ODT was only around 40% of the PZQ reference.

#### Hepatic CYP metabolism

The activity of the drug-metabolising CYPs during PZQ metabolism and the resultant variable exposure of the drug was assessed by multiple studies. With regard to hepatic CYP activity, el Guiniady and colleagues [55] investigated the potential decrease in CYP metabolism in *S*. *mansoni*–infected adult patients with either liver cirrhosis or splenomegaly (data in S6 Table), building on the rodent study by Kokwaro and colleagues [44]. The study concluded that, in general, the C_max_, T_max_, and AUC were significantly higher in the liver-impaired patients compared to the controls. This was determined to be due to the delay in elimination of PZQ to its metabolites as a result of decreased CYP function [55]. In humans, as the severity of cirrhosis increased, the patients had an elevated C_max_ and AUC in comparison to HNV and nonimpaired *S*. *mansoni* patients at the same PZQ dose.

**Fig 4:**
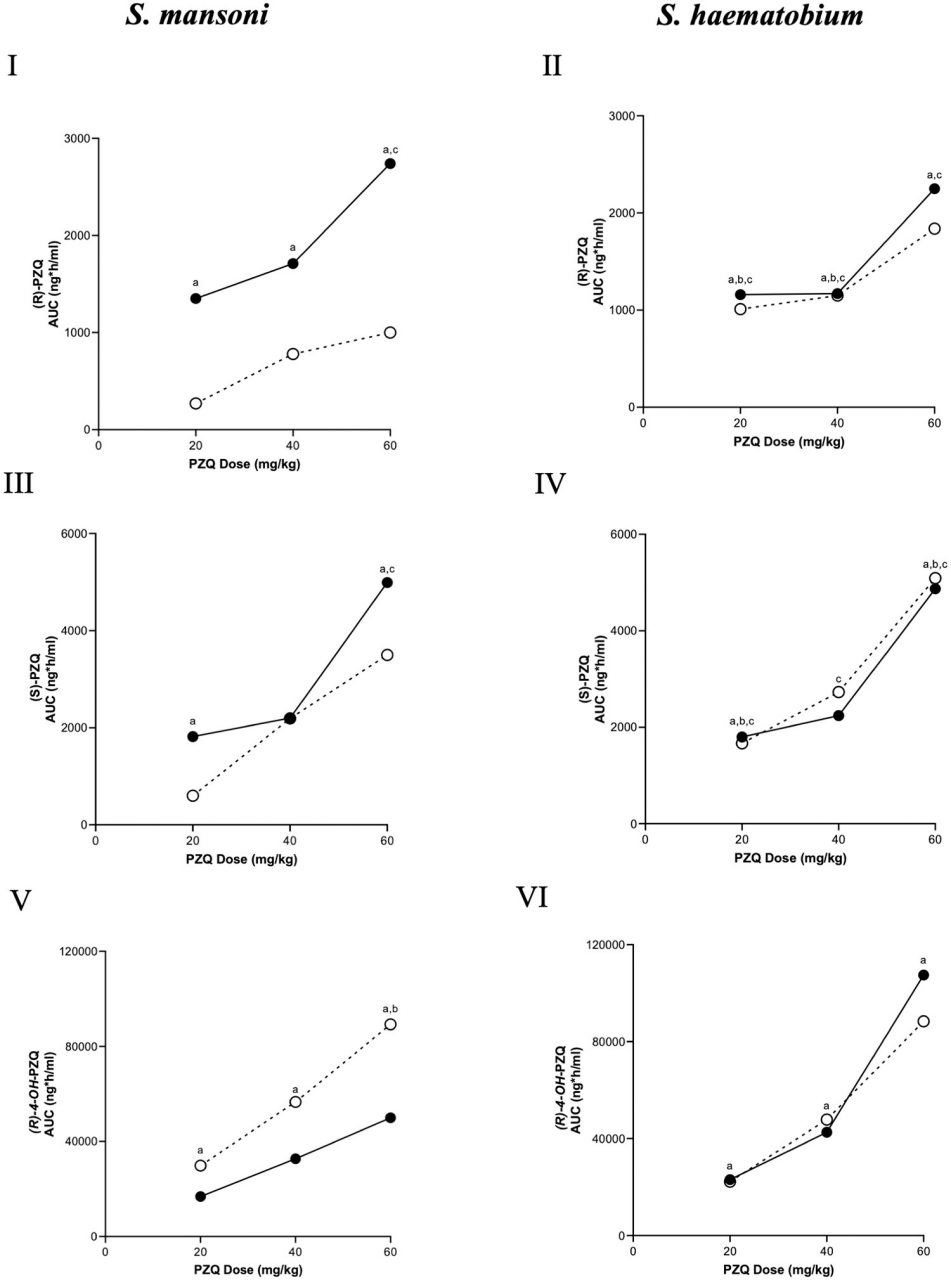
The mean AUC values extracted from the included studies against PZQ dose of (R)-PZQ [I, II], (S)-PZQ [III, IV], and the major metabolite (R)-trans-4-OH-PZQ [V, VI] at 20, 40, and 60 mg/kg. The data are extracted from Kovac and colleagues [56], which investigated *S*. *mansoni* and *S*. *haematobium* infected PSAC (●) and SAC (○) treated with PZQ. The AUC values were then plotted against the dose of PZQ administered for further analysis. *P* < 0.05 was considered statistically significant; a: significant difference between SAC and PSAC for the same dose and analyte, b: significant difference between *S*. *haematobium* and *S*. *mansoni* for the same dose, age group, and analyte, and c: significant difference between (R)-PZQ and (S)-PZQ for the same dose, age group, and species. AUC, area under the curve; PSAC, preschool-aged children; PZQ, praziquantel; SAC, school-aged children.

Concerning the hepatic metabolism of PZQ in children, Kovac and colleagues [56] investigated the PK parameters of SAC and PSAC regarding age-related metabolism and the level of CYP maturity in children. Data points from three papers [56–58] were obtained (S7 Table), including the t_1/2_, T_max_, C_max_, and AUC. The increase in AUC with increased dose for SAC and PSAC infected with *S*. *mansoni* and *S*. *haematobium* were compared in Fig 4, measuring the (R)-PZQ (Fig 4[I, II]), (S)-PZQ (Fig 4[II, IV]), and the major metabolite *(R)-4-OH*-PZQ (Fig 4[V, VI]).

There was a significant difference between the AUC values of the infected *S*. *mansoni* SAC and PSAC at the same dose and analyte, with the exception of (S)-PZQ at 40 mg/kg. Additionally, the AUC of (R)-PZQ and (S)-PZQ in *S*. *mansoni* SAC was found to be significantly different at the same dose but only at 60 mg/kg for PSAC. Generally, the AUC values of the infected *S*. *mansoni* PSAC were higher than the infected *S*. *mansoni* SAC for both the PZQ enantiomers (Fig 4[I, III]) [56], suggesting that once dosed with PZQ, PSAC metabolised the drug more slowly. This is in accordance with the age-related model by Bonate and colleagues [52], which stated that due to the decreased CYP activity of the PSAC, the drug remains in circulation longer, resulting in a higher AUC. Conversely, the AUC for the *(R)-4-OH*-PZQ metabolite was higher in SAC because they had potentially metabolised the drug more rapidly than PSAC (due to increased CYP activity), leading to a higher circulating level of the metabolite (Fig 4[V]). Regarding the exposure in *S*. *haematobium*-infected children, there was no significant difference between exposure levels in *S*. *haematobium*-infected PSAC and SAC (Fig 4[II, IV, VI]).

#### PZQ dosing

In infected children, PZQ is delivered at a dose extrapolated from adult tolerance studies. Bustinduy and colleagues [58] and Bonate and colleagues [52] both described the limitations of current treatment models and further investigated whether the dosing regime was contributing to low CR in children (S8 Table). Both studies highlighted that the extrapolation of infected African children from healthy European adult volunteers is misjudged, and the resultant model cannot predict the differing bioavailability in each population. The resultant extrapolation model concluded that the standard method was not predictive of treatment success and that further studies are required to optimise PZQ treatment, looking at the effect of higher dosage, sex, weight, and PZQ enantiomeric activity [58]. Additionally, Bonate and colleagues [52] described the potential of using a model that includes the degree of maturation of the CYP isoforms involved in PZQ metabolism, and aimed to use this model to predict PZQ exposure (AUC) based on age.

#### Impact of food

There are additional factors which need to be considered that could affect PZQ efficacy, specifically factors not attributed to the host biology. The varying fasting and fed state of multiple studies were found to contribute to variable exposure of PZQ, noting that the bioavailability of PZQ increases with the administration of food [64]. Mandour and colleagues [63] confirmed that, after oral administration in HNV, the bioavailability is reliant on food intake, with PZQ clearance affected by content of the diet. A high oil diet enhanced PZQ absorption, and a high carbohydrate diet appeared to inhibit CYP activities due to an accumulation of metabolites, preventing further metabolism and allowing PZQ to remain in systemic circulation longer.

### Drug–drug interactions

**Fig 5:**
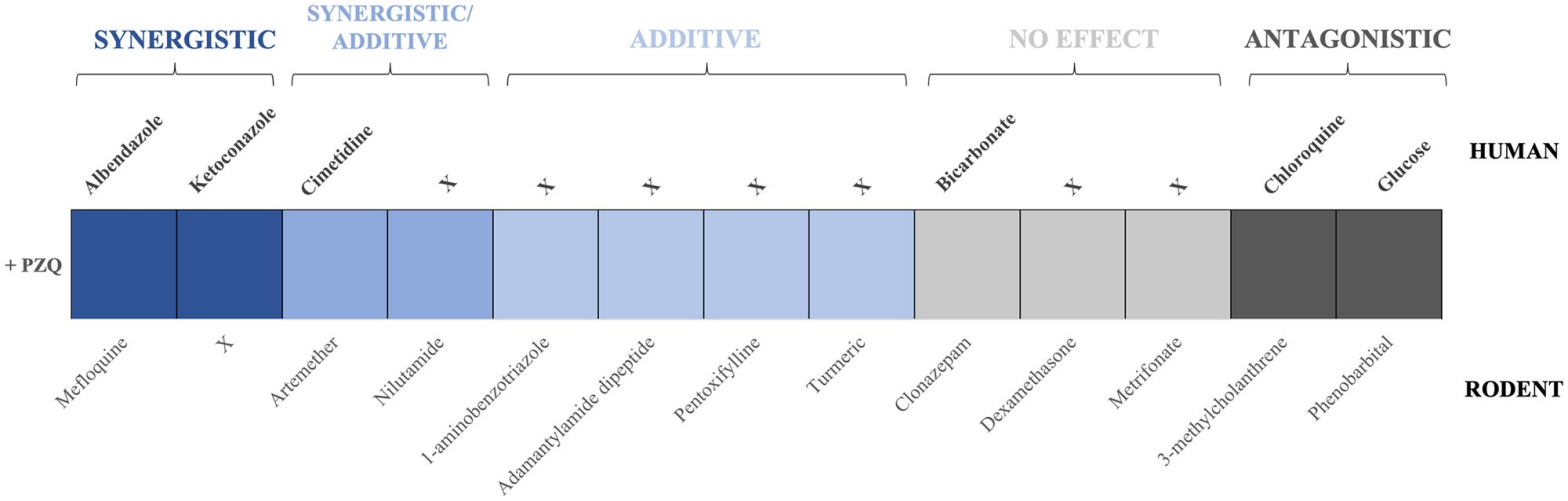
The DDIs identified by the review, and the resultant effect on PZQ exposure. The *in vivo* model and “Drug B” are listed as well as the overall effect of the drug combination on exposure of PZQ. The effect of the drug combination on PZQ efficacy in rodents was calculated based on the percentage change in WBR of each drug alone in comparison to the combined action (S9 Table) [73–83]. The effect of the drug combination on PZQ efficacy in humans was calculated based on the percentage change in AUC of each drug alone in comparison to the combined action (S10 Table) [54, 59, 84–86]. AUC, area under the curve; DDI, drug–drug interaction; PZQ, praziquantel; WBR, worm burden reduction.

There is a paucity of information on DDIs with PZQ, with only 17 published papers on the topic. Of these studies, many evaluated the use of DDIs to lower PZQ dosing regimens for a combined drug treatment. To define the effect of each drug combination, an effect-based strategy was introduced, which followed the principle that if one component alters the ability of PZQ to reach the necessary lethal schistosome concentration via alterations to PZQ metabolism, then this action was designated a pharmacological effect [65]. Therefore, this review used a pharmacokinetic-based assessment of DDIs based on the alteration of CYP activities and potentially the overall efficacy of PZQ treatment [66]. This “bioavailability model” focused on the change in the AUC due to these drug combination effects to aid further discussion of potential DDIs that could alter PZQ efficacy [67, 68]. In combination with the descriptive results of each DDI by the papers reviewed, this allowed a comparison of the potential combined effect of the two drugs (*E*_*PZQ+B*_) to the effects of its individual components (*E*_*PZQ*_ and *E*_*B*_) [69]. The effect of the DDI is expressed as (1) a synergistic effect: the combined therapy has a greater therapeutic effect than each drug alone following the principle E_PZQ_+E_B_≪E_PZQ+B_, where E_PZQ+B_ > 20% change in AUC compared with PZQ alone; (2) an additive effect: similar to each drug used individually, no significant increase in activity E_PZQ_+E_B_≈E_PZQ+B_; (3) an antagonistic effect: interactions that could decrease therapeutic efficacy, in which E_PZQ+B_<-20% change in AUC compared with PZQ alone, or (4) no effect to treatment, these results were not outside the threshold of -20%≤E_PZQ+B_≥20% change in AUC compared with PZQ alone [70–72]. The PZQ drug combinations obtained in this review are listed in Fig 5, depicting the result of the drug on PZQ action as described by the published DDI studies.

### Experimental studies: drug–drug interactions

#### Synergistic and additive drug–drug interactions

To reduce the dose of PZQ while still having an efficacious effect, synergistic activity using the antimalarial mefloquine was explored in rodents by El-Lakkany and colleagues [73]. The WBR of PZQ + mefloquine was over double the value of PZQ alone, indicating a synergistic effect that maintained lethal schistosome PZQ concentrations and that could be applicable to humans treated with both drugs. Furthermore, when PZQ combined with another antimalarial, artemether, synergistic and additive effects were observed in separate rodent studies, even when PZQ was reduced to around a quarter of its recommended dose. A PZQ + artemether study by Utzinger and colleagues [74] concluded that the lower doses used in the combined treatment was safer and more efficacious (with over double the WBR) than PZQ alone and was suggested as a basis for a human clinical trial [74, 75].

Another study aimed to decrease the dose of PZQ, with Keiser and colleagues [76] combining nilutamide with PZQ to increase WBR compared to PZQ alone. It appeared that at low doses the combination had an additive effect on PZQ, with nilutamide obtaining a higher WBR alone. Interestingly, a synergistic effect was seen with PZQ + nilutamide (100 mg/kg + 200 mg/kg) dose, with a 67% increase in the WBR than PZQ alone at that same dose, something that was not observed for nilutamide below 200 mg/kg.

Further additive effects were evaluated by El-Lakkany and colleagues [77] and Botros and colleagues [78], using pentoxifylline and adamantylamide dipeptide in combination with PZQ (S11 Table). Both combinations used a subcurative dose of PZQ in the DDI to evaluate the enhancement of each drug on PZQ’s therapeutic effect, with both combinations showing comparable results to the full dose of PZQ [77, 78]. Abla and colleagues [79] co-dosed PZQ with 1-aminobenzotriazole, a pan-CYP inhibitor, and predicted a reduction in PZQ clearance and in turn increase C_max_ and AUC of the pharmacoactive (R)-PZQ in plasma. Yet, in *S*. *mansoni*–infected mice, 1-aminobenzotriazole was found to have only a small impact on the WBR, only increasing it by approximately 25%, reaching similar levels to PZQ alone (additive) taking into account interanimal variability. This was similar to the combination of turmeric and PZQ [80], in which a significant additive effect was observed, with around a 24% increase in WBR.

#### Antagonistic drug–drug interactions

Masimirembwa [81] and colleagues reported two antagonistic effects with PZQ in rats, as combinations with both phenobarbital and 3-methylcholanthrene decreased the C_max_ and AUC of PZQ, with phenobarbital decreasing PZQ exposure greater than 3-methylcholanthrene.

#### No effect

It is also important to analyse the combinations that have no effect on PZQ efficacy and can, therefore, be confidently co-administered without affecting PZQ’s systemic concentration. Araujo and colleagues [82] and Ebeid and colleagues [83] investigated the combination of PZQ with clonazepam and metrifonate. Both studies concluded that there was no beneficial synergistic or additive action with the combined treatments of PZQ and that any antischistosomal action originated from PZQ alone and not a DDI. Furthermore, dexamethasone, a multiple CYP inducer, was expected to antagonistically decrease PZQ exposure and decrease PZQ efficacy due to an increase in the CYPs available to metabolise PZQ. Contrary to these predictions by Abla and colleagues [79], dexamethasone decreased plasma exposure of (R)-PZQ by approximately 10 fold but did not affect overall PZQ efficacy in rodents.

### Human studies: drug–drug interactions

**Fig 6:**
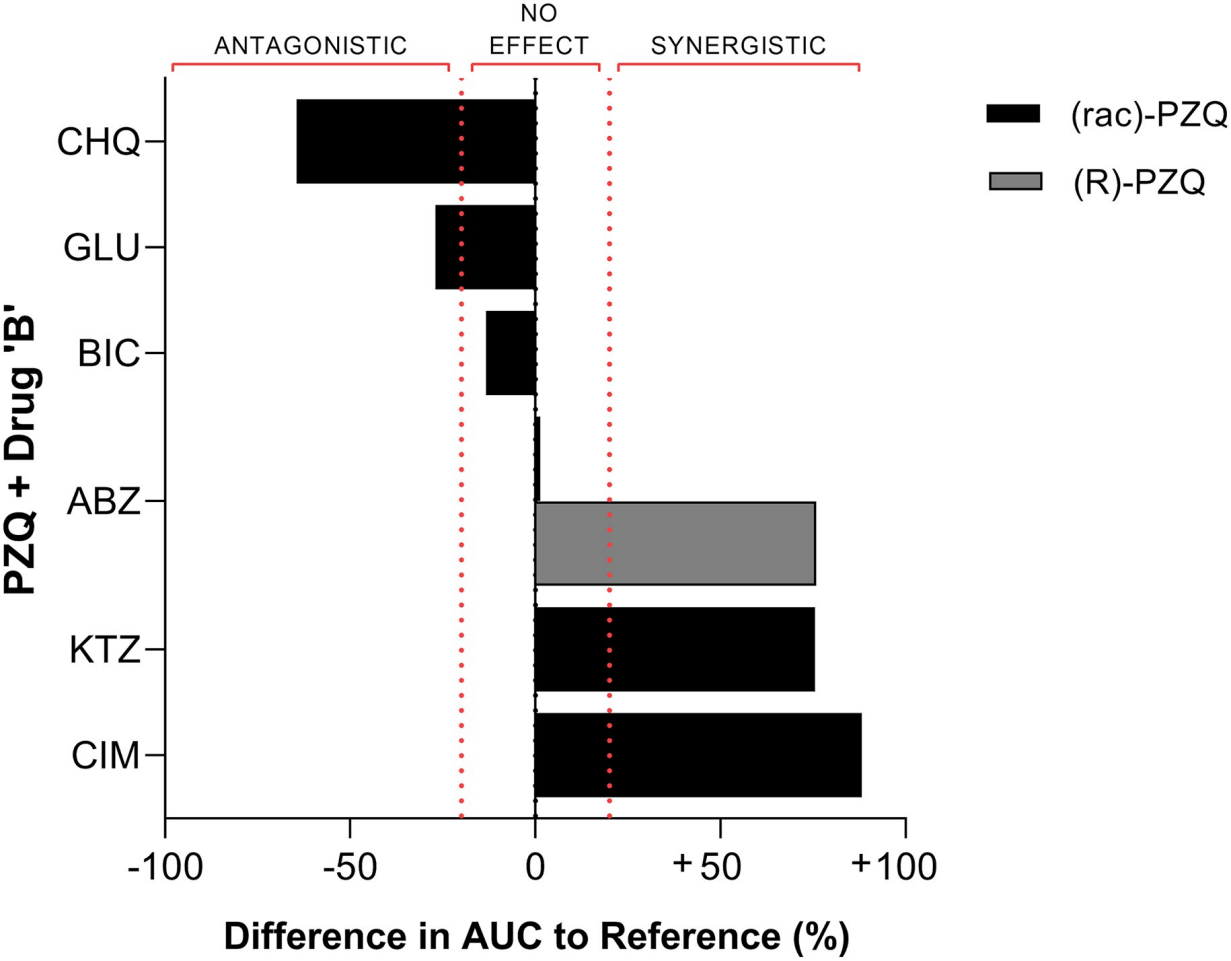
A summary of the human DDIs. Each bar represents the percentage change in AUC during the drug combination in comparison to PZQ alone. The effect of the drug combination is listed above the bar chart, with ±20% representing the boundaries of no effect due to interindividual variation [87]. The data were extracted from five papers [54, 59, 84–86], compiled in S10 Table, analysed to create percentage change in AUC and then plotted to aid further analysis. ABZ, albendazole; AUC, area under the curve; BIC, bicarbonate; CHQ, chloroquine; CIM, cimetidine; DDI, drug–drug interaction; GLUC, glucose; KTZ, ketoconazole; PZQ, praziquantel.

Five studies [54, 59, 84–86] describing human DDIs and the resultant variable drug exposure after oral administration of drug and food combinations were examined. The drug combinations and the percentage change in AUC from PZQ are shown in Fig 6.

#### Synergistic DDIs

Nleya and colleagues [84] evaluated the effect of combining PZQ with ketoconazole, as ketoconazole is a prominent inhibitor of the CYP3A4/5 isoforms which are known metabolic pathways of PZQ [88]. There was a 75% increase in the relative bioavailability of PZQ (measured by the change in the AUC) when co-dosed with ketoconazole, with the C_max_ increasing by 96% in response to the DDI. To measure the extent of the synergistic effect, the study was dosed at one-half the recommended value for a PZQ treatment (20 mg/kg PZQ + 200mgs ketoconazole). Despite this, 9 of 29 individuals reached the 1μM therapeutic threshold required to kill adult schistosomes, compared to only 2 of 29 when PZQ was given alone.

PZQ + cimetidine were evaluated by Jung and colleagues [59], with the potential to simultaneously treat schistosomiasis and neurocysticercosis, building on a study by Metwally and colleagues [85] that noted elevated PZQ sera concentrations with this DDI. Compared to PZQ alone, the plasma levels of PZQ more than doubled during combined drug administration, with a C_max_ greater than 400 ng/ml even after 12 hours, suggesting a synergistic effect.

Lima and colleagues [54] treated HNV with a combination of PZQ and albendazole (anthelmintic used for treating soil-transmitted helminths), with the aim of improving the therapeutic efficacy of both drugs by increasing plasma concentrations of the active forms of both drugs. This conclusion can be visualised in Fig 6, in which the combination of PZQ + albendazole appears to have no effect on the AUC of rac-PZQ. Opposing this, Lima and colleagues [54] showed that PZQ increased the C_max_ of the active albendazole metabolite, and, in turn, albendazole increased the AUC of the pharmacologically active (R)-PZQ: a synergistic effect. In Fig 6, the AUC of (R)-PZQ increased by 76%, which would not be apparent if only the rac-PZQ was measured.

#### Antagonistic drug–drug interactions

Masimirembwa and colleagues [86] investigated the antagonistic effect of the CYP inhibitor chloroquine in HNV, with chloroquine expected to increase the bioavailability of PZQ due to its action as a CYP3A4 inhibitor. In contrast, it was found to decrease the C_max_ and AUC of PZQ, decreasing overall exposure, with 50% of individuals studied not reaching the lethal schistosome plasma concentration of 1μM. Masimirembwa and colleagues [86] determined this effect was not due to alterations in CYP activities but, rather, an alternate mechanism of chloroquine.

An antagonistic effect was also observed by Metwally and colleagues [85], when PZQ was co-dosed with glucose. However, as depicted in Fig 6, the percentage change of the AUC for the combination compared to PZQ alone was −27%, which was just outside of the 20% “no effect” threshold [87]. Therefore, although this has been noted as an antagonistic effect with glucose, it may be due to interindividual variation slightly skewing the results because there were only 20 participants in this study, and there may be no effect regarding this interaction.

#### No effect

Although the combination of bicarbonate with PZQ did reduce the AUC of PZQ [85], it was not outside the 20% threshold (see Fig 6). Similar to glucose, this combination is not likely to have an effect on the efficacy of PZQ and is most likely due to the mean calculation of the AUC and the interindividual variation in the metabolism of the participants in the study.

### Pharmacogenetics

#### Human studies: pharmacogenetics

The search did not yield any studies targeting the pharmacogenetics of PZQ metabolism. However, the data extracted from the included studies contained numerous examples of metabolic variability in the human studies. Although there were no specific studies on pharmacogenetics, the reasoning behind the variable drug exposure and efficacy of PZQ was postulated by many authors to be attributed to interindividual variation and host genetic factors [43, 45, 55–59, 61–63, 75, 85, 86]. The majority of the included studies did not evaluate this hypothesis further but merely stated that CYP polymorphisms and CYP-related maturity may be causing variation in PZQ PK parameters and bioavailability. The ethnicities of the individuals included in the human studies in this review were also recorded. Of the 17 human studies, 17.6% studied individuals from Europe, 17.6% from South America, and 64.7% from Africa. The ethnicities of the study populations included in this review were recorded to aid further discussion.

### Metabolite analysis of praziquantel

**Fig 7:**
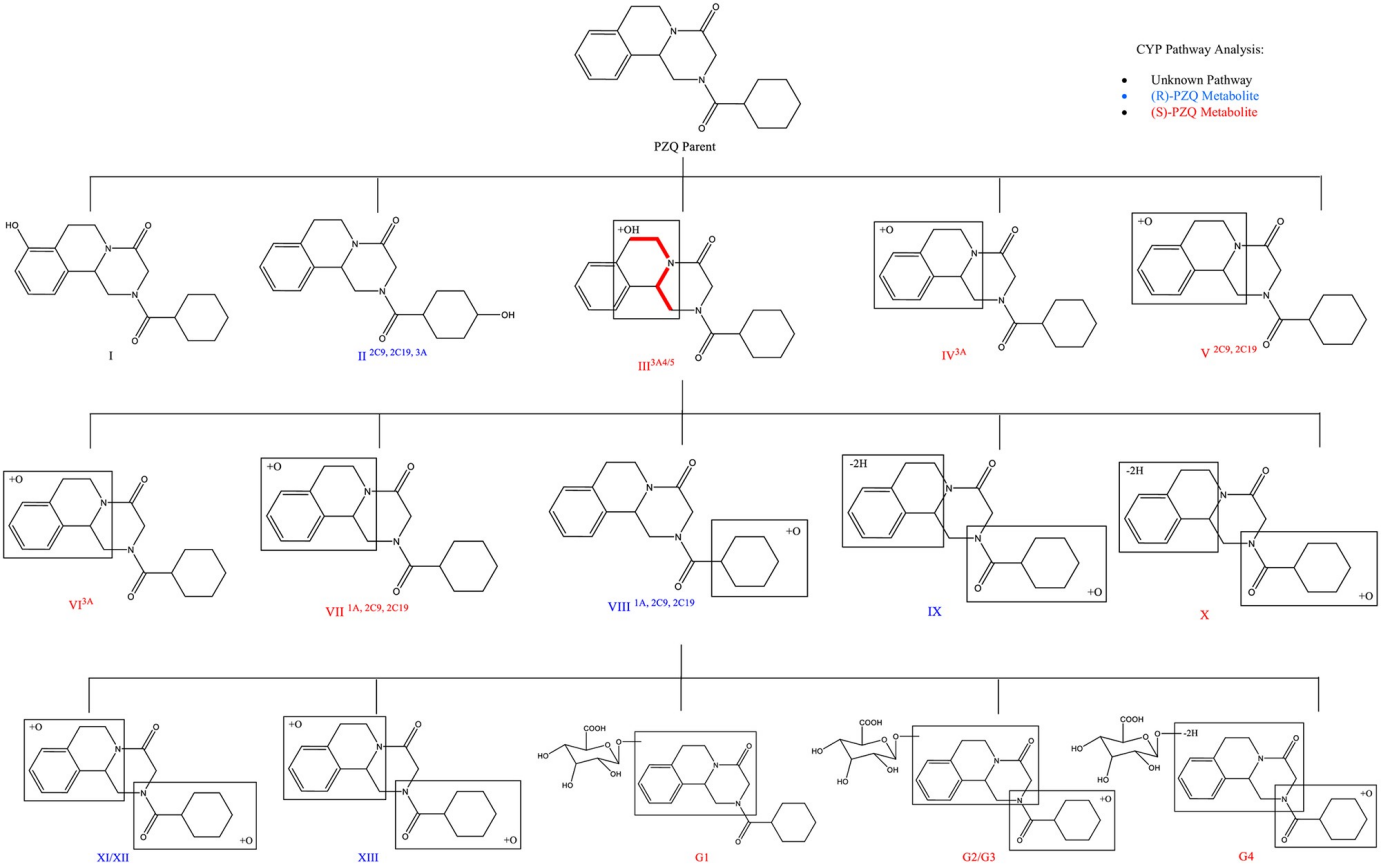
A comprehensive map of PZQ metabolites extracted and combined from studies that focused on the metabolite analysis of PZQ. I: 8-OH-PZQ [89], II: 4-OH-PZQ [28, 53], III: X-OH-PZQ [84], IV: O-PZQ [28], V:O-PZQ [28], VI: O-PZQ [28], VII: O-PZQ [28], VIII: O-PZQ [28], IX: (-2H)-O-PZQ [28], X: (-2H)-O-PZQ [28], XI/XII: O_2_-PZQ [28], XIII: O_2_-PZQ [28], G1: Gluc-PZQ [28], G2/G3: Gluc-O-PZQ [28], G4: (-2H)-Gluc-O-PZQ [28]. The chemical structures and metabolite map were created using ChemDraw Prime v16.0. Gluc, glucuronide; PZQ, praziquantel.

Seventeen metabolites were elucidated from four papers [28, 53, 84, 89], and their structures are displayed in Fig 7. PZQ and its enantiomeric metabolites, including their CYP pathway and structure, were evaluated by multiple *in vivo* and *in vitro* techniques. Using human plasma, human urine, human and mouse liver microsomes, and human recombinant enzymes, there were 8 distinguishable mono-oxidised metabolites, 2 dehydrogenated mono-oxidised metabolites, 3 di-oxidised metabolites, and 4 glucuronide metabolites detected.

**Fig 8:**
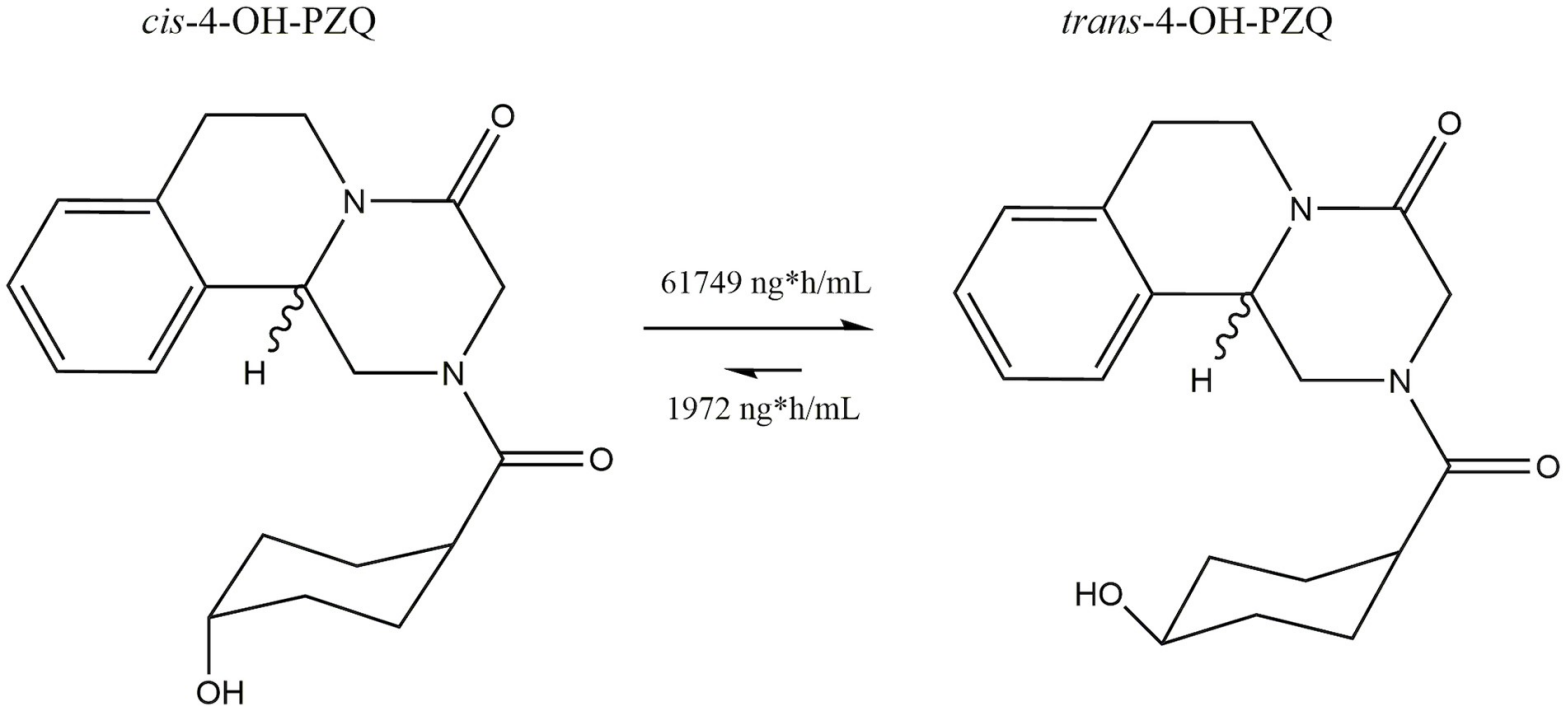
The favourable formation of *trans*-4-OH-PZQ compared to the *cis*-4-OH-PZQ isomer created from pharmacokinetic data by Nleya and colleagues [84]. The equatorial position points upwards and the axial position points downwards. The chemical structures were created using ChemDraw Prime v16.0. PZQ, praziquantel.

A study by Nleya and colleagues [84] depicted the metabolic pathway of PZQ to its main metabolite; 4-OH-PZQ (Fig 7[II]). This study showed that when PZQ was concomitantly administered with CYP3A4/5 inhibitor ketoconazole, significant changes in the level of metabolites in circulation were observed. The AUC of the main 4-OH-PZQ metabolites were increased by 57% (*cis-*) and 67% (*trans-*) when co-dosed with ketoconazole, with 30 times more *trans* isomer than the *cis* as seen in Fig 8. The higher levels of the *trans*- isomer stems from the favourable cyclohexane ring conformation placing the bulky OH substituent equatorial to prevent steric hindrance compared to the axial (*cis*-) conformation [90].

## Discussion and conclusion

Heterogeneity in PZQ drug efficacy has been reported in multiple studies [18, 19]. Given the concern about the development of drug resistance in schistosome parasites, there has been considerable research on the potential for parasites developing resistance. However, it is critical to investigate the impact of nonparasite related factors that give rise to variability in PZQ drug efficacy. The majority of studies determined this to be due to interindividual variation and host genetic factors with no further analysis [43, 45, 55–59, 61–63, 75, 85, 86]. Here, we determined whether variable cure rates in humans could be attributed to fluctuating levels of PZQ in systemic circulation and explored the factors that influence an individual’s response to PZQ treatment, focusing on the metabolism of the drug and the resultant efficacy of treatment.

Multiple studies reviewed confirmed (R)-PZQ as the pharmacologically active enantiomer and that the exposure of the desired (R)-PZQ enantiomer was significantly lower than the (S)-PZQ when dosed as rac-PZQ [46, 49–54]. The reasoning behind the chiral differences in the elimination of each enantiomer has been postulated to be due the variations in their affinity in binding to the active site of the CYPs [28]. If the affinity of (R)-PZQ for CYP1A2 is higher than (S)-PZQ, then the metabolism of the active (R)-PZQ to its major metabolite (as described by Nleya and colleagues [84]) would be greater than metabolism of (S)-PZQ, resulting in a more rapid decrease in (R)-PZQ plasma exposure than for (S)-PZQ. However, as affinity to CYP1A2 has not yet been evaluated, the metabolic differences between (*R*)-PZQ and (*S*)-PZQ are still unclear. These studies highlight the benefits of treatment with the (R)-PZQ enantiomer alone in comparison to using the racemate and supporting the development of new formulations with the active enantiomer, as is the case for the ODT paediatric formulations of PZQ currently undergoing clinical trials [91]. In this case, the ODT formulation increased the AUC of the desired (R) enantiomer for the rac-ODT and (R)-ODT in comparison to the reference rac-PZQ, but it had a nonproportional PK profile [51, 52]. This limited the interpretation of each PZQ enantiomers PK profile in the ODT and emphasises the need for an (R)-ODT dose-finding study in PSAC to fully define the PK in paediatric patients and find the correct dosing regimen.

In experimental models, host schistosome infection status was an important factor influencing PZQ’s pharmacokinetic parameters, with PZQ’s exposure parameters in infected animals higher on average than in uninfected animals [42–45]. The studies proposed that *S*. *mansoni* infection interferes with liver function, preventing PZQ metabolism. However, this does not easily translate to human hosts. Instead variable exposure in human hosts tended to be associated with alterations in the liver’s capacity to metabolise PZQ due to other factors, as observed in studies in which liver disease impaired CYP function, leading to increased levels of PZQ in systemic circulation. In patients suffering from liver cirrhosis and also treated with PZQ, the C_max_ and AUC increased with the severity of cirrhosis in comparison to nonimpaired *S*. *mansoni*–infected and healthy patients at the same PZQ dose [55]. The increase in exposure parameters is contrary to the study performed in rats by Kokwaro and colleagues [44] and further highlights the limitations of experimental models in capturing human dynamics.

In human studies, age-related maturation of hepatic CYPs has been postulated, suggesting that the hepatic CYP pathways are not fully matured in children and affect drug AUC [52, 58]. The effect of age-related metabolism was investigated in a study of *S*. *mansoni*–infected children in which the AUC values of the PSAC were higher than the SAC for both the PZQ enantiomers in the circulation, yet the AUC for the *(R)-4-OH*-PZQ metabolite was higher in SAC [56]. This suggested PSAC could be metabolising the drug more slowly than the SAC, with higher concentrations of the parent drug found in PSAC and higher concentrations of metabolite found in SAC. This is consistent with higher exposure to active PZQ in the PSAC, resulting in higher cure rates compared to SAC. This is contrary to a study performed by Coulibaly and colleagues in which the cure rates in PSAC were lower at 40 and 60 mg/kg than in the SAC. This concurred with the data from this review at 60 mg/kg, as the SAC had a 5% higher cure rate than PSAC. However, Coulibaly and colleagues did suggest that during the study the crushing of the tablets may have altered the PK of PZQ, which may account for the difference in efficacy [92]. This age-related effect has not yet been observed in *S*. *haematobium*. Ofori-Adjei and colleagues [57] reported no differences in the PK parameters between uninfected and *S*. *haematobium* infected SAC, concluding that *S*. *haematobium* infection does not influence PZQ metabolism due to alterations to hepatic function [56].

Non–host-related factors also affected exposure and efficacy of PZQ treatment, and these included PZQ brands and the coadministration of food or other drugs. Simultaneous administration of PZQ and food can alter the PK as food can delay absorption, affect stomach pH, alter blood flow, or interact with the PZQ itself [93]. One of the initial PZQ tolerance studies in humans provided the adult volunteers with a standardised meal for continuity in the study [61], but during PCT programs, this is not particularly representative, as diet can vastly differ. Fed groups have a lower clearance of PZQ due to altered absorption, highlighting the importance of attaining a fed state during treatment and in human PK studies to enhance success of exposure to the anthelmintic [63]. Of the different brands of PZQ on the market, Distocide and Biltricide were found to be more comparable in terms of exposure to pure PZQ [42]. Even so, the PZQ brands evaluated showed a decreased AUC, C_max_, and t_1/2_ and, therefore, decreased efficacy and drug exposure in comparison to pure PZQ, indicating that treatment failures may be due to variable exposure of PZQ arising from the quality of the PZQ formulation.

A host attribute that can also affect drug metabolism is genetic polymorphism of liver enzymes involved in drug metabolism, i.e., pharmacogenetics. While CYP pharmacogenetic studies have been conducted in different ethnic populations [94], there are relatively few studies on PZQ metabolism. [94]. In recent publications, trials of more efficacious formulations and age-dosing models were being performed in European populations instead of the majority drug target population in Africa [52, 61]. Although 90% of schistosomiasis infected people live in Africa there have been no studies of PZQ population genetics in Africa [95, 96], highlighting the need for pharmacogenetic studies on the clinically relevant CYP variants in target populations [94]. For example, CYP2D6 is a metabolic route of PZQ; therefore, the presence of CYP2D6*17, which is unique to populations of African origin [97], exemplifies the need for its impact on PZQ metabolism and overall treatment efficacy to be evaluated. Nleya and colleagues [84] have provided evidence CYP1A2 may be the metabolic route of PZQ to its main metabolite in Zimbabwean volunteers. As a recent study reported that Zimbabwean children in schistosomiasis-endemic areas exhibited decreased CYP1A2 activity, and, as PZQ is metabolised via this pathway, this could also have detrimental implications on bioavailability [45].

In order to translate molecular findings into drug metabolism and predict efficacy, there is a need to characterise the PZQ metabolites, their quantities and their effect. Wang and colleagues [28] have performed the most comprehensive evaluation of PZQ’s metabolites, albeit in experimental models, which identified phase I and phase II metabolites using both *in vivo* and *in vitro* methods. Fifteen metabolites were structurally identified from urine and faeces of mice 24 hours after PZQ dosing. The *in vitro* incubations using human liver microsomes (HLM) and human recombinant enzymes confirmed the metabolic activity of CYP1A2, CYP2C9, 2C19, 2D6, and 3A4/5, with metabolic products identified via all of these pathways. However, the *in vivo* results from the experimental studies were not identical to the human *in vitro* HLM, with 3 dioxidised metabolites not detected in HLM. The Zimbabwean study, one of the few human studies, also characterised the metabolic pathway of PZQ to its main metabolite: 4-OH-PZQ [84]. The combination of PZQ with CYP3A4/5 inhibitor ketoconazole resulted in significant changes in the AUC of the main 4-OH-PZQ metabolites, with increases of 57% (*cis-*) and 67% (*trans-*) when co-dosed with ketoconazole. The overall increase in the AUC of both 4-OH-PZQ metabolites revealed that when the CYP3A4/5 pathway was inhibited, there was a greater exposure of the main metabolite in the circulation. This indicates that CYP3A4/5 is not the metabolic route of 4-OH-PZQ and suggests that other CYP pathways, primarily CYP1A2 or CYP2C19, are instead involved in the principal elimination of the active parent drug. So what metabolite is the CYP3A4/5 pathway producing? The study reported a novel metabolite, X-OH-PZQ, which was suppressed upon administration of ketoconazole with PZQ, and therefore appeared to be dependent on CYP3A4/5 for its formation. The X-OH-PZQ levels were reduced by approximately 57%, providing categorical evidence that this novel metabolite is produced via the CYP3A4/5 pathway. Other studies have discussed the use of CYP3A inhibitors to reduce the conversion of PZQ to its main metabolite (Fig 7[II]) [53]; however, based on the results from this study, it appears that the introduction of a CYP1A2/CYP2C19 inhibitor to PZQ may result in a greater exposure of active parent drug. If the 4-OH-PZQ metabolic pathway is inhibited, then a reduction in the elimination of PZQ could occur and result in higher circulating concentration of the parent drug enhancing therapeutic efficacy. The exact structure of X-OH-PZQ was, at this time, not yet fully determined; to achieve this, the exact nature of the hydroxylation biotransformation must be evaluated. The intricacies of metabolite identification is potentially one of the factors contributing to the limited number of results addressing this topic, as Schepmann and colleagues [89] demonstrated, dedicating an entire paper to elucidating the structure of one phase I metabolite: 8-OH-PZQ (Fig 7[I]). By obtaining further information on PZQ’s metabolites via the detection and quantification of each metabolite, they could be compared between individuals for variability. As individual genetic variation in the CYP enzymes could affect PZQ metabolite concentration in systemic circulation, there is the potential to use the metabolite itself as genetic marker without the need for sequencing.

As schistosomiasis is coendemic with several other parasites and pathogens, affected populations can be subjected to drug coadministration, giving rise to DDIs. Malaria is one such co-endemic parasite, and coadministering the antimalarials mefloquine and artemether with PZQ was investigated in experimental models, building on work from an *in vitro* experiment by Keiser and colleagues [98]. The coadministration of reduced doses of PZQ + mefloquine and PZQ + artemether showed enhanced pharmacological activity and efficacy over PZQ alone [73–75]. Other coendemic infections include intestinal helminths, and helminth control programs often coadminister PZQ and albendazole or mebendazole [99]. In humans, the combination of the anthelmintic albendazole + PZQ resulted in an increase in the plasma concentrations of the active enantiomers of both drugs [54]. Previous reports presented contradictory data regarding the DDIs between PZQ and albendazole, concluding that coadministration of PZQ + albendazole does not alter PZQ PK [100, 101]. However, Lima and colleagues [54] demonstrated a synergistic interaction upon this combination with the AUC of the (R)-PZQ increasing by 76%, which would mean an increase in the exposure of the active drug to the schistosomes. This interaction was determined to be due to the albendazole inhibiting CYP1A and CYP3A pathways, indicating the (R)-PZQ cannot be metabolised and remains in systemic circulation longer, leading to higher (R)-PZQ plasma concentrations. Nonetheless, even with the advantage of increased PZQ exposure, the increased plasma concentrations of both active drugs risks unknown adverse effects without further investigation [54]. This beneficial combination is not the case with chloroquine. This antimalarial drug, when coadministered with PZQ, decreased the AUC of PZQ by approximately 64% compared to PZQ alone [86] and may create too low a systemic concentration of PZQ to be lethal to schistosomes. This antagonistic effect on PZQ metabolism was deemed not to be due to CYP alterations, highlighting the need for further investigations into this DDI mechanism.

Other drugs affecting CYP activity have also been shown to synergistically affect PZQ plasma availability. For example, cimetidine more than doubled the plasma levels of PZQ during combined drug administration than PZQ alone. Cimetidine nonselectively inhibits CYP1A2, 2C9, 2D6, and 3A4 [102], all of which are metabolic pathways of PZQ. A nilutamide + PZQ combination was also found to be synergistic in rodent infections, significantly increasing WBR by 67% compared with PZQ alone [76]. In humans, coadministering PZQ + ketoconazole can increase the duration of active PZQ exposure [84] by inhibiting the CYP3A4/5 pathway to reduce clearance of PZQ by 30% and enhance parasite exposure to PZQ [84]. The AUC value at 20 mg/kg PZQ when coadministered with ketoconazole was comparable with a study using 40 mg/kg PZQ alone [63]. Despite this, upon a linear extrapolation of the level of PZQ exposure from this combination, it predicted only a 37.5% reduction in PZQ dose. Therefore, regardless of this synergistic activity, there would not be a significant reduction of the current tablet size with this DDI and, so, would not be a better alternative than the development of a new formulation [84].

There is definite need for more studies of PZQ metabolism in humans, specifically in affected populations. The majority of studies reviewed here were experimental models of schistosomiasis, and findings from these cannot always be easily translated or extrapolated to human hosts, particularly the PZQ metabolite characterisation studies. Although animal studies are useful as models of potential DDIs, some enzymes that are orthologous to the CYPs are not representative of human responses [43]. A methodological limitation of this review was the measurement used. One of the main measures of exposure used to compare drug exposure in this review is the AUC; however, the absolute bioavailability is generally a more representative parameter. It is calculated using the AUC of the intravenous route and the AUC of the IV dose [103], but this data was not available for the majority of included PK studies. The quantity and type of data available in the original publications also limited the conclusions in this review, particularly when data were presented as the mean values postanalysis and not the raw data; therefore, no further statistical analysis was possible. Additionally, we could not identify clearly established drug-specific thresholds beyond which reductions in AUC might lead to alterations in drug effect. The use of the 20% threshold in change in AUC was a representative value to identify important changes in systemic bioavailability [87]. While the lack of defined thresholds may hinder the clinical interpretations in this review, there was no definitive method to determine the DDIs from the data available. Therefore, the DDI effects described here were based on efficacy of PZQ treatment stemming from exposure to the active drug and are not necessarily the confirmation of a mechanistic interaction. Also, the low number of published studies that were obtained for each topic using the search criteria highlighted the gap in the knowledge in PZQ metabolism during schistosomiasis treatment. When the search criteria used in this review was applied to tuberculosis, there was approximately eight times the number of search results (S12 Table). This increase in results could be due to the number of different drugs used in tuberculosis, in comparison to the one drug used for schistosomiasis; nonetheless, this shows the difference in the quantity of published research for schistosomiasis in comparison to a disease that can occur in every part of the world.

Overall, schistosomiasis control is predominantly reliant on a single drug, PZQ, for treating millions of people. Variable cure rates from the drug raise concerns about the possibility of the development of drug resistance amongst the parasites; therefore, there is a need to determine the sources of heterogeneity in cure rates and determine the relative contribution of host-related factors. Our review has shown that several such factors can result in variable levels of PZQ in systemic circulation that potentially contribute to these low cure rates. These include drug formulation (enantiomers) and brand, the health of the host’ s liver, host age, coadministered drugs, and host genetics. There is need for more of these studies in affected human populations, especially in Africa, where host and parasite attributes are studied simultaneously, to fully understand the sources of heterogeneity in PZQ efficacy.

## Supporting information

S1 Fig Flow chart guides by the PRISMA for a systematic review, including the records excluded based on the specific criteria. PRISMA, Preferred Reporting Items for Systematic Reviews and Meta-Analysis.
(TIFF)

S1 Table Included studies obtained using the inclusion and exclusion criteria of this review, separated into topics of focus. (PDF)

S2 Table Comparison of different brands of PZQ on the PK parameters in a rodent model. PK, pharmacokinetic; PZQ, praziquantel.
(PDF)

S3 Table Comparison of different brands of PZQ on efficacy of treatment in an infected murine model. PZQ, praziquantel.
(PDF)

S4 Table Comparison of the WBR in an infected murine model when dosing racemic PZQ, or a single enantiomer. PZQ, praziquantel; WBR, worm burden reduction.
(PDF)

S5 Table Pharmacokinetic parameters of PZQ enantiomers and its major metabolites in HNV after administration of racemic PZQ, or the single PZQ enantiomer itself. HNV, healthy normal volunteers; PZQ, praziquantel.
(PDF)

S6 Table Pharmacokinetic parameters in human models after administration of racemic PZQ; includes P and HNV. HNV, healthy normal volunteers; P, infected patients; PZQ, praziquantel.
(PDF)

S7 Table Pharmacokinetic parameters of PZQ, PZQ enantiomers, and the major metabolite in HSAC, infected SAC, and infected PSAC after the administration of racemic PZQ. HSAC, healthy school age children; PSAC, preschool-aged children; PZQ, praziquantel; SAC, school-aged children.
(PDF)

S8 Table Efficacy parameters of racemic PZQ treatment on infected PSAC and SAC, and the effect of the different brands of PZQ available. PSAC, preschool-aged children; PZQ, praziquantel; SAC, school-aged children.
(PDF)

S9 Table The comparison of dosing racemic PZQ alone, or with drug "B" on PZQ’s antischistosomal efficacy in rodent models. PZQ, praziquantel.
(PDF)

S10 Table The pharmacokinetic parameters of PZQ, its enantiomers, and the major metabolite in HNV after the administration of racemic PZQ with drug "B" to evaluate potential DDIs. DDI, drug–drug interactions; HNV, healthy normal volunteers; PZQ, praziquantel.
(PDF)

S11 Table The comparison of racemic PZQ alone, and with drug "B" in reducing hepatic granuloma diameter in murine models. PZQ, praziquantel.
(PDF)

S12 Table Comparison of the results of this systematic review in comparison to tuberculosis. (PDF)
