# Supplementary figures and images for: Drug metabolism and pharmacokinetics of praziquantel: A review of variable drug exposure during schistosomiasis treatment in human hosts and experimental models
Source: PLoS Negl Trop Dis. 2020 Sep 25;14(9):e0008649. doi: 10.1371/journal.pntd.0008649 (PMC7518612; doi:10.1371/journal.pntd.0008649)

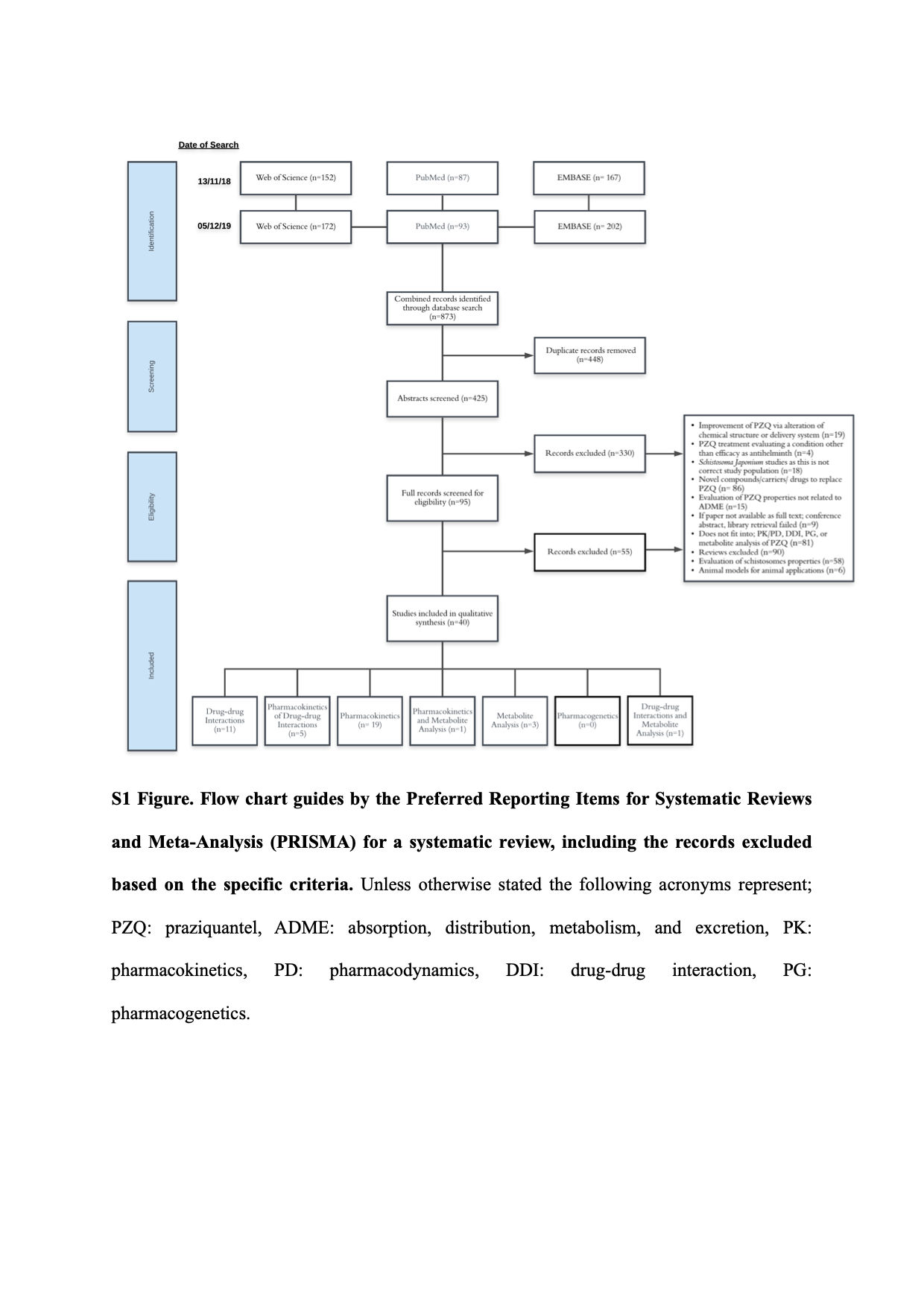

Supplement: S1 Fig — PRISMA, Preferred Reporting Items for Systematic Reviews and Meta-Analysis. (TIFF) [file pntd.0008649.s001.tiff]
