# Supplementary material for: Drug metabolism and pharmacokinetics of praziquantel: A review of variable drug exposure during schistosomiasis treatment in human hosts and experimental models
Source: PLoS Negl Trop Dis. 2020 Sep 25;14(9):e0008649. doi: 10.1371/journal.pntd.0008649 (PMC7518612; doi:10.1371/journal.pntd.0008649)
Supplement: S1 Table — (PDF) [file pntd.0008649.s002.pdf]

**S1 Table. Included studies obtained using the inclusion and exclusion criteria of this review, separated into topics of focus.** The inclusion ID was designated, A: Pharmacokinetics/Pharmacodynamics**,** B: Drug-drug interactions**,** C: Metabolite analysis**,** D: Pharmacogenetics

| Inclusion ID | Title |
|---|---|
| A/B | Abla, N., et al. (2017). "Evaluation of the pharmacokinetic-pharmacodynamic relationship of praziquantel in the schistosoma mansoni mouse model: Possible clinical implications." Tropical Medicine and International Health 22 (Supplement 1): 136-137. |
| B | Araujo, N., et al. (2008). "Association of oxamniquine praziquantel and clonazepam in experimental Schistosomiasis mansoni." Memorias Do Instituto Oswaldo Cruz 103(8): 781-785. |
| A | Bonate, P. L., et al. (2018). "Extrapolation of praziquantel pharmacokinetics to a pediatric population: a cautionary tale." Journal of Pharmacokinetics and Pharmacodynamics 45(5): 747-762. |
| A | Botros, S., et al. (2011). "Comparative efficacy and bioavailability of different praziquantel brands." Experimental Parasitology 127(2): 515-521. |
| B | Botros, S., et al. (2006). "Effect of adamantylamide dipeptide as adjuvant therapy to praziquantel in mice infected with different S. mansoni isolates." Apmis 114(7-8): 480-491. |
| A | Botros, S. S., et al. (2006). "Drug-metabolizing enzymes and praziquantel bioavailability in mice harboring Schistosoma mansoni isolates of different drug susceptibilities." Journal of Parasitology 92(6): 1344-1349. |
| B | Botros, S. S., et al. (2010). "Praziquantel efficacy in mice infected with PZQ non-susceptible S. mansoni isolate treated with artemether: Parasitological, biochemical and immunohistochemical assessment." Apmis 118(9): 692-702. |
| A | Bustinduy, A. L., et al. (2016). "Population Pharmacokinetics and Pharmacodynamics of Praziquantel in Ugandan Children with Intestinal Schistosomiasis: Higher Dosages Are Required for Maximal Efficacy." Mbio 7(4). |
| B | Campelo, Y. D. M., et al. (2017). "Synergistic effects of in vitro combinations of piplartine, epiisopiloturine and praziquantel against Schistosoma mansoni." Biomedicine & Pharmacotherapy 88: 488-499. |
| B | Ebeid, F. A. (1995). "Efficacy of a combination of metrifonate and praziquantel in the treatment of schistosoma-mansoni infection in albino mice." Arzneimittel-Forschung/Drug Research 45-1(4): 519-521. |
| A | Elguiniady, M. A., et al. (1994). "Clinical and pharmacokinetic study of praziquantel in Egyptian schistosomiasis patients with and without liver cell failure." American Journal of Tropical Medicine and Hygiene 51(6): 809-818. |
| B | El-Lakkany, N. and M. Nosseir (2007). "Pharmacodynamics of pentoxifylline and/or praziquantel in murine schistosomiasis mansoni." Apmis 115(3): 184-194. |
| B | El-Lakkany, N. M., et al. (2011). "Pharmacodynamics of mefloquine and praziquantel combination therapy in mice harbouring juvenile and adult Schistosoma mansoni." Memorias Do Instituto Oswaldo Cruz 106(7): 814-822. |
| A | Gotardo, M. A., et al. (2011). "Modulation of expression and activity of cytochrome P450s and alteration of praziquantel kinetics during murine schistosomiasis." Memorias Do Instituto Oswaldo Cruz 106(2): 212-219. |
| B | Hussein, A., et al. (2017). "Evaluation of the Anti-schistosomal Effects of Turmeric (Curcuma longa) Versus Praziquantel in Schistosoma mansoni Infected Mice." Iranian Journal of Parasitology 12(4): 587-596. |
| A/B | Jung, H., et al. (1997). "Pharmacokinetic study of praziquantel administered alone and in combination with cimetidine in a single-day therapeutic regimen." Antimicrobial Agents and Chemotherapy 41(6): 1256-1259. |
| B | Keiser, J., et al. (2011). "Interactions of mefloquine with praziquantel in the schistosoma mansoni mouse model and in vitro." Journal of Antimicrobial Chemotherapy 66(8): 1791-1797. |

| | |
|---|---|
| B | Keiser, J., et al. (2010). "Activity of antiandrogens against juvenile and adult Schistosoma mansoni in mice." Journal of Antimicrobial Chemotherapy 65(9): 1991-1995. |
| A | Kokwaro, G. O. and G. Taylor (1990). "Effect of experimentally-induced hepatic cirrhosis on the pharmacokinetics of orally administered praziquantel in the rat." Eur J Drug Metab Pharmacokinet 15(3): 199-202. |
| A | Kovac, J., et al. (2018). "Pharmacokinetics of Praziquantel in Schistosoma mansoni- and Schistosoma haematobium-Infected School- and Preschool-Aged Children." Antimicrobial Agents and Chemotherapy 62(8). |
| A | Kovac, J., et al. (2018). "Evaluation of a novel micro-sampling device, Mitra (TM), in comparison to dried blood spots, for analysis of praziquantel in Schistosoma haematobium-infected children in rural Cote d'Ivoire." Journal of Pharmaceutical and Biomedical Analysis 151: 339-346. |
| A | Leopold, G., et al. (1978). "Clinical pharmacology in normal volunteers of praziquantel, a new drug against schistosomes and cestodes. An example of a complex study covering both tolerance and pharmacokinetics." European Journal of Clinical Pharmacology 14(4): 281-291. |
| A/B | Lima, R. M., et al. (2011). "Albendazole-praziquantel interaction in healthy volunteers: kinetic disposition, metabolism and enantioselectivity." British Journal of Clinical Pharmacology 71(4): 528-535. |
| A/C | Lima, R. M., et al. (2009). "Enantioselective analysis of praziquantel and trans-4-hydroxypraziquantel in human plasma by chiral LC-MS/MS: Application to pharmacokinetics." Journal of Chromatography B-Analytical Technologies in the Biomedical and Life Sciences 877(27): 3083-3088. |
| A | Mandour, M. E., et al. (1990). "Pharmacokinetics of praziquantel in healthy volunteers and patients with schistosomiasis." Transactions of the Royal Society of Tropical Medicine and Hygiene 84(3): 389-393. |
| A/B | Masimirembwa, C. M., et al. (1993). "Effects of phenobarbital and 3-methylcholanthrene pretreatment on the pharmacokinetics of praziquantel in rats." European Journal of Drug Metabolism and Pharmacokinetics 18(3): 261-264. |
| A/B | Masimirembwa, C. M., et al. (1994). "The effect of chloroquine on the pharmacokinetics and metabolism of praziquantel in rats and in humans." Biopharmaceutics & Drug Disposition 15(1): 33-43. |
| A | Meister, I., et al. (2014). "Activity of praziquantel enantiomers and main metabolites against Schistosoma mansoni." Antimicrobial Agents and Chemotherapy 58(9): 5466-5472. |
| A | Metwally, A., et al. (1995). "Impact of drug dosage and brand on bioavailability and efficacy of praziquantel." Pharmacological Research 31(1): 53-59. |
| A/B | Metwally, A., et al. (1995). "Effect of cimetidine, bicarbonate and glucose on the bioavailability of different formulations of praziquantel." Arzneimittel-Forschung/Drug Research 45-1(4): 516-518. |
| A | Ofori-Adjei, D., et al. (1988). "Oral praziquantel kinetics in normal and Schistosoma haematobium-infected subjects." Ther Drug Monit 10(1): 45-49. |
| A | Pehrson, P. O., et al. (1983). "Treatment with praziquantel in a patient with schistosomiasis and chronic renal failure." Trans R Soc Trop Med Hyg 77(5): 687-688. |
| C | Schepmann, D. and G. Blaschke (2001). "Isolation and identification of 8-hydroxypraziquantel as a metabolite of the antischistosomal drug praziquantel." J Pharm Biomed Anal 26(5-6): 791-799. |
| A | Tanaka, M., et al. (1989). "A comparison of the antischistosomal effect of levo- and dextro-praziquantel on Schistosoma japonicum and S. mansoni in mice." American Journal of Tropical Medicine and Hygiene 41(2): 198-203. |
| B | Utzinger, J., et al. (2001). "Effect of combined treatment with praziquantel and artemether on Schistosoma japonicum and Schistosoma mansoni in experimentally infected animals." Acta Tropica 80(1): 9-18. |
| C | Wang, H. N., et al. (2014). "Metabolic profiling of praziquantel enantiomers." Biochemical Pharmacology 90(2): 166-178. |
| A | Zhang, D. L., et al. (2017). "A quantification method for determination of racemate praziquantel and R-enantiomer in rat plasma for comparison of their pharmacokinetics." Journal of Chromatography B-Analytical Technologies in the Biomedical and Life Sciences 1048: 64-69. |
