# Supplementary material for: Drug metabolism and pharmacokinetics of praziquantel: A review of variable drug exposure during schistosomiasis treatment in human hosts and experimental models
Source: PLoS Negl Trop Dis. 2020 Sep 25;14(9):e0008649. doi: 10.1371/journal.pntd.0008649 (PMC7518612; doi:10.1371/journal.pntd.0008649)
Supplement: S2 Table — PK, pharmacokinetic; PZQ, praziquantel. (PDF) [file pntd.0008649.s003.pdf]

S2 Table. Comparison of different brands of praziquantel (PZQ) on the pharmacokinetic (PK) parameters in a rodent model. The brands, when named, were listed in subscript; P: Pure PZQ, T3A: T3A Pharmaceuticals Company PZQ, BILT: Biltricide, DIS: Distocide, BILH: Bilharzid, EPI: Epiquantel. The superscript descriptions show the additional information of the study. R: Rat, HR: Healthy Rat, M: Mouse, HM: Healthy Mouse, PB-PZQ: Polymorph B, (a): PID30 (male), (b): PID30 (female), (c): PID55 (male), (d): PID55 (female), (e): Pre-dosed with THIO and in a fasted state, (f): PZQ-susceptible S. mansoni isolate CD, (g): PZQ-non-susceptible S. mansoni isolate EE2 and BANL. Unless otherwise stated the following acronyms represent; AUC: Area under the plasma concentration-time curve, B ($AUC_{0\text{-}inf}$): Area under the plasma concentration-time curve extrapolated from time zero to infinity, $t_{1/2}$: Elimination half-life, C_{max}: Peak plasma concentration, T_{max}: Time to reach peak plasma concentration following drug administration.

**Drug Administered**		**Drug Measured**	**PZQ Dose (mg/kg)**	**N**	**$t_{1/2}$ (1/h)**	**T_{max} (h)**	**C_{max} (ng/ml)**	**AUC (ng/ml/h)**
PZQ [1]			10					
	HR	*PZQ*		6	1.54	0.58	377	441
	HR	*(R)- PZQ*		6	1.08	0.5	168	177
PZQ [2]			25					
	R (e)	*PZQ*		5	0.55	0.65	900	-
PZQ [3]			50					
	M) (mansoni)	*(R)- PZQ*		4	5.9	0.5	700	2200^{B} (ng*h/mL)
	M) (mansoni)	*(S)- PZQ*		4	5.5	0.5	200	3600^{B} (ng*h/mL)
PB-PZQ								
	M) (mansoni)	*(R)- PZQ*		4	5.4	0.5	200	400^{B} (ng*h/mL)
	M) (mansoni)	*(S)- PZQ*		4	5.4	0.5	100	300^{B} (ng*h/mL)
PZQ [3]			100					
	M) (mansoni)	*(R)- PZQ*		4	2.6	0.5	700	1300^{B} (ng*h/mL)
	M) (mansoni)	*(S)- PZQ*		4	0.4	0.5	100	300^{B} (ng*h/mL)
PB-PZQ [3]								
	M) (mansoni)	*(R)- PZQ*		4	10.6	0.5	300	1900^{B} (ng*h/mL)
	M) (mansoni)	*(S)- PZQ*		4	20.4	0.5	100	1600^{B} (ng*h/mL)
PZQ [3, 4]		*PZQ*	200					
	M (a) (mansoni)			7		0.46	31598	19650
	M (b) (mansoni)			7		0.64	34168	40583
	M (c) (mansoni)			7		0.4	26433	81017
	M (d) (mansoni)			7		0.63	27473	76833
	M) (mansoni)	*(R)- PZQ*		4	3.3	0.8	2100	7100^{B} (ng*h/mL)

M [j] (mansoni)	*(S)- PZQ*		4	6	0.8	2900	11500 [B] (ng*h/mL)
PB-PZQ [3]							
M [j] (mansoni)	*(R)- PZQ*		4	3.1	0.5	900	
M [j] (mansoni)	*(S)- PZQ*		4	1.5	0.5	400	3000 [B] (ng*h/mL)
							1000 [B] (ng*h/mL)
PZQ [3]		300					
M [j] (mansoni)	*(R)- PZQ*		4	5.9	0.5	800	2500 [B] (ng*h/mL)
M [j] (mansoni)	*(S)- PZQ*		4	6.4	0.5	300	1000 [B] (ng*h/mL)
PB-PZQ [3]							
M [j] (mansoni)	*(R)- PZQ*		4	4	0.8	900	3100 [B] (ng*h/mL)
M [j] (mansoni)	*(S)- PZQ*		4	5.4	0.8	200	900 [B] (ng*h/mL)
PZQ [3, 4]	*PZQ*	400					
M [(a)] (mansoni)			7		0.4	35996	57700
M [(b)] (mansoni)			7		0.64	58458	114383
M [(c)] (mansoni)			7		0.4	31846	132800
M [(d)] (mansoni)			7		0.63	51399	146017
M [j] (mansoni)	*(R)- PZQ*		4	3.3	0.8	2700	6400 [B] (ng*h/mL)
M [j] (mansoni)	*(S)- PZQ*		4	4.7	0.8	500	1900 [B] (ng*h/mL)
PB-PZQ [3]							
M [j] (mansoni)	*(R)- PZQ*		4	5.3	0.5	1600	3100 [B] (ng*h/mL)
M [j] (mansoni)	*(S)- PZQ*		4	7.1	0.5	700	1500 [B] (ng*h/mL)
PZQ [5]	*PZQ*	500					
M [(f)] (mansoni)			77	1.11	0.087	33330	42920
M [(g)] (mansoni)			77	0.89	0.084	23440	26990
M [(g)] (mansoni)			77	0.93	0.076	18600	11280
PZQ [6]							
HM (P)			11	1.05	0.11	14840	12260
HM (T3A)			11	0.46	0.12	10630	8350
HM(DIS)			11	0.78	0.08	12790	10190
HM (BILT)			11	1.18	0.11	13210	11100
HM *(BILH)*			11	0.42	0.08	11000	6620
HM *(EPI)*			11	0.52	0.09	10790	8290

References

1. Zhang DL, Wang HN, Ji JB, Nie L, Sun DQ. A quantification method for determination of racemate praziquantel and R-enantiomer in rat plasma for comparison of their pharmacokinetics. Journal of Chromatography B-Analytical Technologies in the Biomedical and Life Sciences. 2017;1048:64-9. doi: 10.1016/j.jchromb.2017.02.013. PubMed PMID: WOS:000397686500008.
2. Kokwaro GO, Taylor G. Effect of experimentally-induced hepatic cirrhosis on the pharmacokinetics of orally administered praziquantel in the rat. European journal of drug metabolism and pharmacokinetics. 1990;15(3):199-202. Epub 1990/07/01. doi: 10.1007/bf03190204. PubMed PMID: 2253649.
3. Lombardo FC, Perissutti B, Keiser J. Activity and pharmacokinetics of a praziquantel crystalline polymorph in the Schistosoma mansoni mouse model. European Journal of Pharmaceutics and Biopharmaceutics. 2019;142:240-6. doi: https://doi.org/10.1016/j.ejpb.2019.06.029.
4. Gotardo MA, Hyssa JT, Carvalho RS, De-Carvalho RR, Gueiros LS, Siqueira CM, et al. Modulation of expression and activity of cytochrome P450s and alteration of praziquantel

kinetics during murine schistosomiasis. Memorias Do Instituto Oswaldo Cruz. 2011;106(2):212-9. doi: 10.1590/s0074-02762011000200016. PubMed PMID: WOS:000289961100016.
5. Botros SS, El-Din SHS, El-Lakkany NM, Sabra ANA, Ebeid FA. Drug-metabolizing enzymes and praziquantel bioavailability in mice harboring Schistosoma mansoni isolates of different drug susceptibilities. Journal of Parasitology. 2006;92(6):1344-9. doi: 10.1645/ge-865r.1. PubMed PMID: WOS:000243959800032.
6. Botros S, El-Lakkany N, el-Din SHS, Sabra AN, Ibrahim M. Comparative efficacy and bioavailability of different praziquantel brands. Experimental parasitology. 2011;127(2):515-21. doi: 10.1016/j.exppara.2010.10.019. PubMed PMID: WOS:000286643300029.
