# Supplementary material for: Drug metabolism and pharmacokinetics of praziquantel: A review of variable drug exposure during schistosomiasis treatment in human hosts and experimental models
Source: PLoS Negl Trop Dis. 2020 Sep 25;14(9):e0008649. doi: 10.1371/journal.pntd.0008649 (PMC7518612; doi:10.1371/journal.pntd.0008649)
Supplement: S3 Table — PZQ, praziquantel. (PDF) [file pntd.0008649.s004.pdf]

S3 Table. Comparison of different brands of praziquantel (PZQ) on efficacy of treatment in an infected murine model. Statistical significance was assessed by Botros *et al.* [1] using a two-tailed Student's t-test between the means of different groups. Results for this study were considered significant if P< 0.05: *P<0.05, **P<0.01, ***P<0.001. The brands, when named, are listed in subscript; P: Pure PZQ, T3A: T3A PZQ, BILT: Biltricide, DIS: Distocide, BILH: Bilharzid, EPI: Epiquantel. The superscript descriptions show the additional information of the study; (a): PZQ-susceptible S. mansoni isolate CD, (b): PZQ-non-susceptible S. mansoni isolate EE2. Unless otherwise stated the following acronyms represent; M: Mouse, WBR: Worm burden reduction.

Drug Administered	Drug Measured	PZQ Dose (mg/kg)	N	WBR (%)
PZQ $_{(P)}$ [1]	*PZQ*	(5x25)		
M $^{(a)}$ (mansoni)			9	53.81
M $^{(b)}$ (mansoni)			7	47.4
PZQ $_{(T3A)}$ [1]				
M $^{(a)}$ (mansoni)			8	39.9
M $^{(b)}$ (mansoni)			8	31.9**
PZQ $_{(DIS)}$ [1]				
M $^{(a)}$ (mansoni)			7	44.8
M $^{(b)}$ (mansoni)			8	33.3
PZQ $_{(BILT)}$ [1]				
M $^{(a)}$ (mansoni)			7	49.3
M $^{(b)}$ (mansoni)			9	38.9
PZQ $_{(BILH)}$ [1]				
M $^{(a)}$ (mansoni)			7	40.8
M $^{(b)}$ (mansoni)			8	23.2**
PZQ $_{(EPI)}$ [1]				
M $^{(a)}$ (mansoni)			7	41.25
M $^{(b)}$ (mansoni)			7	28.1*

References

1. Botros S, El-Lakkany N, el-Din SHS, Sabra AN, Ibrahim M. Comparative efficacy and bioavailability of different praziquantel brands. Experimental parasitology. 2011;127(2):515-21. doi: 10.1016/j.exppara.2010.10.019. PubMed PMID: WOS:000286643300029.
