# Supplementary material for: Drug metabolism and pharmacokinetics of praziquantel: A review of variable drug exposure during schistosomiasis treatment in human hosts and experimental models
Source: PLoS Negl Trop Dis. 2020 Sep 25;14(9):e0008649. doi: 10.1371/journal.pntd.0008649 (PMC7518612; doi:10.1371/journal.pntd.0008649)
Supplement: S4 Table — PZQ, praziquantel; WBR, worm burden reduction. (PDF) [file pntd.0008649.s005.pdf]

S4 Table. Comparison of the WBR in an infected murine model when dosing racemic praziquantel (PZQ), or a single enantiomer. The relationship between WBR and drug formulation was found to be significantly different (P<0.05) for each PZQ dose based on the results of the Independent-Samples Kruskal-Wallis Test. Unless otherwise stated the following acronyms represent; M: Mouse and WBR: Worm burden reduction.

Drug Administered	Drug Measured	PZQ Dose (mg/kg)	N	WBR (%)
PZQ [1, 2]	*PZQ*			
M (mansoni)		50	5	12.6
M (mansoni)		100	4	15
M (mansoni)		400	6	94.1
M (mansoni)		500	7	38.9
(R)-PZQ [1, 2]	*(R)- PZQ*			
M (mansoni)		50	7	6.8
M (mansoni)		100	6	52
M (mansoni)		200	6	98.1
M (mansoni)		400	3	100
M (mansoni)		500	7	32.4
(S)-PZQ [1, 2]	*(S)- PZQ*			
M (mansoni)		50	6	25.5
M (mansoni)		400	4	18
M (mansoni)		500	7	50.9
M (mansoni)		800	6	19.6

References

1. Meister I, Ingram-Sieber K, Cowan N, Todd M, Robertson MN, Meli C, et al. Activity of praziquantel enantiomers and main metabolites against Schistosoma mansoni. Antimicrobial agents and chemotherapy. 2014;58(9):5466-72. doi: http://dx.doi.org/10.1128/AAC.02741-14. PubMed PMID: 373772543.
2. Tanaka M, Ohmae H, Utsunomiya H, Nara T, Irie Y, Yasuraoka K. A comparison of the antischistosomal effect of levo- and dextro-praziquantel on Schistosoma japonicum and S. mansoni in mice. American Journal of Tropical Medicine and Hygiene. 1989;41(2):198-203. PubMed PMID: 19224762.
