# Supplementary material for: Drug metabolism and pharmacokinetics of praziquantel: A review of variable drug exposure during schistosomiasis treatment in human hosts and experimental models
Source: PLoS Negl Trop Dis. 2020 Sep 25;14(9):e0008649. doi: 10.1371/journal.pntd.0008649 (PMC7518612; doi:10.1371/journal.pntd.0008649)
Supplement: S5 Table — HNV, healthy normal volunteers; PZQ, praziquantel. (PDF) [file pntd.0008649.s006.pdf]

**S5 Table. Pharmacokinetic parameters of praziquantel (PZQ) enantiomers and its major metabolites in in Healthy Normal Volunteers (HNV) after administration of racemic PZQ, or the single PZQ enantiomer itself.** Using an Independent-Samples Kruskal-Wallis Test, the difference in AUC of the enantiomers found to be statistically significant (P=0.007) at 40 mg/kg only. The brands, when named, are listed in subscript: (ODT): represents new racemic 150mg oral dispersion formulation (dispersed in water), L-ODT: (R)-PZQ formulation, CYS: Cysticide formulation given with water. The superscript descriptions show the additional information of the study; (a): Fasting, (b): Without $H_2O$, (c): Crushed tablets and Fed state. Unless otherwise stated the following acronyms represent; AUC: Area under the plasma concentration-time curve, B ($AUC_{0\text{-inf}}$): Area under the plasma concentration-time curve extrapolated from time zero to infinity, $t_{1/2}$: Elimination half-life, $C_{max}$: Peak plasma concentration, $T_{max}$: Time to reach peak plasma concentration following drug administration.

| PZQ Dose (mg/kg) | Drug Administered | Drug Measured | N | $t_{1/2}$ (h) | $T_{max}$ (h) | $C_{max}$ (ng/ml) | AUC | |
|---|---|---|---|---|---|---|---|---|
| | | | | | | | (ng*h/ml) | (ng*h/ml) |
| | *(R)*-PZQ $_{(L\text{-}ODT)}$ [1, 2] | | | | | | | |
| 10 | *HNV* | *(R)-PZQ* | 18 | 1.06 (N=17) | 2.25 | 90 | | 217$^{B}$ (N=17) |
| | *(R)*-PZQ $_{(L\text{-}ODT)}$ [1, 2] | | | | | | | |
| | *HNV* | *(R)-PZQ* | 36 | 2.98 | 2.50 | 379 | | 825$^{B}$ |
| | *HNV* $^{(a)}$ | | 35 | 2.80 | 2.00 | 131 | | 506$^{B}$ |
| 20 | *HNV* $^{(b)}$ | | 36 | 2.71 | 4.00 | 471 | | 955$^{B}$ |
| | PZQ $_{(ODT)}$ [1, 2] | | | | | | | |
| | *HNV* | *(R)-PZQ* | 15 | 1.92 (N=14) | 4.00 | 155 | | 345$^{B}$ |
| | *HNV* | *(S)-PZQ* | 15 | 3.96 | 3.50 | 786 | | 2226$^{B}$ |
| 30 | *(R)*-PZQ $_{(L\text{-}ODT)}$ [1, 2] | | | | | | | |
| | *HNV* | *(R)-PZQ* | 17 | 3.30 | 3.00 | 1052 | | 2324.9$^{B}$ |
| | PZQ $_{(ODT)}$ [1, 2] | | | | | | | |
| | *HNV* | *(R)-PZQ* | 30 | 3.31 | 3.00 | 881 | | 1928,1970$^{B}$ |
| | *HNV* $^{(a)}$ | | 14 | 4.17 | 4.25 | 189 | | 864 ,925$^{B}$ |
| | *HNV* | *(S)-PZQ* | 30 | 4.58 | 3.00 | 2330 | | 7542$^{B}$ |
| | *HNV* $^{(a)}$ | | 14 | 5.28 | 4.50 | 839 | | 4996$^{B}$ |
| 40 | PZQ $_{(CYS)}$ [1, 2] | | | | | | | |
| | *HNV* | *(R)-PZQ* | 30 | 3.83 | 1.50 | 763 | | 2048$^{B}$, 1994 |
| | *HNV* $^{(c)}$ | | 15 | 3.07 (N=14) | 2.50 | 446 | | 1538$^{B}$ (N=14),1343 |
| | *HNV* | *(S)-PZQ* | 30 | 4.62 | 2.00 | 2179 | | 8293$^{B}$ |
| | *HNV* | | 15 | 4.35 | 3.50 | 1556 | | 6509$^{B}$ |
| | PZQ $_{(ODT)}$ [1, 2] | | | | | | | |
| 60 | *HNV* | *(R)-PZQ* | 15 | 4.20 | 3.00 | 1548 | | 4871$^{B}$,4770 |
| | *HNV* | *(S)-PZQ* | 15 | 4.35 | 4.00 | 3234 | | 14686$^{B}$ |

| | | | | | | | | |
|---|---|---|---|---|---|---|---|---|
| | PZQ [3, 4] | | | | | | | |
| | | *HNV* | *(R)-PZQ* | 1 | 1.68 | 2.73 | 51 | 338[B] |
| | | *HNV* | | 9 | 1.55 | 2.67 | 160 | 870[B] |
| | | *HNV* | *(S)-PZQ* | 1 | 1.33 | 2.33 | 165 | 857[B] |
| **1500** | | *HNV* | | 9 | 1.46 | 2.55 | 520 | 2990[B] |
| | | *HNV* | *(R)-4-OH-PZQ* | 1 | 1.51 | 2.56 | 2176 | 12865[B] |
| | | *HNV* | | 9 | 1.7 | 2.72 | 1310 | 8800[B] |
| | | *HNV* | | 1 | 1.68 | 3.32 | 1169 | 7717[B] |
| | | *HNV* | *(S)-4-OH-PZQ* | 9 | 1.91 | 3.05 | 780 | 5600[B] |

## References


1. Bonate PL, Wang TL, Passier P, Bagchus W, Burt H, Lupfert C, et al. Extrapolation of praziquantel pharmacokinetics to a pediatric population: a cautionary tale. Journal of Pharmacokinetics and Pharmacodynamics. 2018;45(5):747-62. doi: 10.1007/s10928-018-9601-1. PubMed PMID: WOS:000445761600008.
2. Bagchus WM, Bezuidenhout D, Harrison-Moench E, Kourany-Lefoll E, Wolna P, Yalkinoglu O. Relative Bioavailability of Orally Dispersible Tablet Formulations of Levo- and Racemic Praziquantel: Two Phase I Studies. Clinical and translational science. 2019;12(1):66-76. Epub 2018/12/12. doi: 10.1111/cts.12601. PubMed PMID: 30536632; PubMed Central PMCID: PMCPMC6342245.
3. Lima RM, Ferreira MAD, Carvalho T, Fernandes BJD, Takayanagui OM, Garcia HH, et al. Albendazole-praziquantel interaction in healthy volunteers: kinetic disposition, metabolism and enantioselectivity. British journal of clinical pharmacology. 2011;71(4):528-35. doi: 10.1111/j.1365-2125.2010.03874.x. PubMed PMID: WOS:000288249500008.
4. Lima RM, Ferreira MAD, Ponte TMD, Marques MP, Takayanagui OM, Garcia HH, et al. Enantioselective analysis of praziquantel and trans-4-hydroxypraziquantel in human plasma by chiral LC-MS/MS: Application to pharmacokinetics. Journal of Chromatography B-Analytical Technologies in the Biomedical and Life Sciences. 2009;877(27):3083-8. doi: 10.1016/j.jchromb.2009.07.036. PubMed PMID: WOS:000270246400021.
