# Supplementary material for: Drug metabolism and pharmacokinetics of praziquantel: A review of variable drug exposure during schistosomiasis treatment in human hosts and experimental models
Source: PLoS Negl Trop Dis. 2020 Sep 25;14(9):e0008649. doi: 10.1371/journal.pntd.0008649 (PMC7518612; doi:10.1371/journal.pntd.0008649)
Supplement: S6 Table — HNV, healthy normal volunteers; P, infected patients; PZQ, praziquantel. (PDF) [file pntd.0008649.s007.pdf]

S6 Table. Pharmacokinetic parameters in human models after administration of racemic praziquantel (PZQ); includes Infected Patients (P) and Healthy Normal Volunteers (HNV). The brands, when named, were listed in subscript: (ODT): represents new racemic 150mg oral dispersion formulation (dispersed in water), BILT: Biltricide, DIS: Distocide, CYS: Cysticide formulation given with water. The superscript descriptions show the additional information of the study (a): Class A liver cirrhosis, (b): Class B liver cirrhosis, (c): Class C liver cirrhosis, (d): Fasting, (e): African population, (f): High oil content, (g): Low oil content, (h): Uraemic Patient. Unless otherwise stated the following acronyms represent; AUC: Area under the plasma concentration-time curve, A (AUC_{0-t}): Area under the plasma concentration-time curve from time zero to time t, B (AUC_{0-inf}): Area under the plasma concentration-time curve extrapolated from time zero to infinity, $t_{1/2}$: Elimination half-life, C_{max}: Peak plasma concentration, T_{max}: Time to reach peak plasma concentration following drug administration.

Drug Administered	PZQ Dose (mg/kg)	N	$t_{1/2}$ (h)	T_{max} (h)	C_{max} (ng/ml)	AUC	
						(ng/ml/h)	(ng*h/ml)
PZQ [1]	5						
HNV		6	1.70	2.75	48		167[A]
PZQ [1]	10						
HNV		6	1.30	2.58	66		209[A]
PZQ [1-4]	20						
HNV		6	1.18	2.08	250		645[A]
HNV$_{\text{(BILT)}}$ [(e)]		10	0.97	1.65	846	1303, 3550	
HNV$_{\text{(DIS)}}$ [(e)]		10	1.24	2.60	558	2133, 1563	
HNV$_{\text{(ODT)}}$		15	3.78	3.50	950		2569[B]
PZQ [2, 4-7]	40						
HNV$_{\text{(BILT)}}$		1	1.28	2.75	1200	10890	
HNV$_{\text{(BILT)}}$		1	1.98	2.00	5000	25040	
HNV$_{\text{(BILT)}}$		1	1.44	2.00	2100	9840	
HNV$_{\text{(BILT)}}$		1	2.57	1.50	500	2760	
HNV$_{\text{(BILT)}}$		1	2.67	2.00	2400	13290	
HNV$_{\text{(BILT)}}$		1	1.98	1.25	100	2300	
HNV$_{\text{(BILT)}}$		1	4.30	1.75	4400	27600	
HNV$_{\text{(BILT)}}$		1	1.60	2.25	3600	18100	
HNV$_{\text{(BILT)}}$		8	1.93	1.89	2130	11750	
HNV$_{\text{(BILT)}}$		10	2.18	2.00	1281	1303	
HNV$_{\text{(BILT)}}$ [(d)]		6	6.30	1.60	823	2110[B]	
HNV$_{\text{(BILT)}}$ [(d)]		6	2.30	2.60	978	3823[B]	
P$_{\text{(BILT)}}$ [(h)] *(haematobium)*		1		1.00	1040		
HNV$_{\text{(CYS)}}$		30	4.41	1.75	2974		10447
HNV$_{\text{(CYS)}}$		15	4.20	2.50	2040		8092
HNV$_{\text{(CYS)}}$		36	3.79	2.50	727		2066
HNV$_{\text{(DIS)}}$		10	2.78	1.72	685	1562	
HNV$_{\text{(DIS)}}$ [(d)]		6	8.90	2.10	249	15928[B]	
HNV$_{\text{(DIS)}}$ [(d)]		8	3.30	2.60	978	4089[B]	
HNV$_{\text{(DIS)}}$ [(f)]		8	2.50	2.70	1570	5699[B]	
HNV$_{\text{(DIS)}}$ [(g)]		8	2.30	2.00	2093	7126[B]	
P$_{\text{(DIS)}}$ [(a)] *(mansoni)*		10		1.37	931	3870	
P$_{\text{(DIS)}}$ [(b)] *(mansoni)*		10		2.21	1469	10720	
P$_{\text{(DIS)}}$ [(c)] *(mansoni)*		10		3.20	3573	45350	
P$_{\text{(DIS)}}$ [(d, e)] *(mansoni)*		9	11.90	1.60	1618	5409[B]	
P$_{\text{(DIS)}}$ *(mansoni)*		10		1.48	833	3023	
HNV$_{\text{(ODT)}}$		30	4.46	3.00	3250		9627[B]
HNV$_{\text{(ODT)}}$ [(d)]		14	4.89	4.50	1042		5956[B]
PZQ [1]	50						
HNV		8	1.19	1.88	1319		3931[A]
PZQ [4]	60						
HNV$_{\text{(ODT)}}$		15	4.32	3.5	4780		19679.5[B]
PZQ [1, 8]							
HNV	(3 x 10)	6	0.84	2.25	65		
HNV	(3 x 20)	6	1.22	1.08	479		
HNV	(3 x 25)	6	1.30	2.25	344		
HNV	(3 x 25)	1	1.70			5870	
HNV	(3 x 25)	1	2.70			11930	
HNV	(3 x 25)	1	1.70			7850	
HNV	(3 x 25)	1	1.70			2720	
HNV	(3 x 25)	1	2.10			8620	
HNV	(3 x 25)	1	1.80			7490	
HNV	(3 x 25)	1	1.20			7410	
HNV	(3 x 25)	1	1.80			12340	

References

1. Leopold G, Ungethum W, Groll E, Diekmann HW, Nowak H, Wegner DH. Clinical pharmacology in normal volunteers of praziquantel, a new drug against schistosomes and cestodes. An example of a complex study covering both tolerance and pharmacokinetics. European journal of clinical pharmacology. 1978;14(4):281-91. Epub 1978/12/01. PubMed PMID: 729622.
2. Metwally A, Bennett J, Botros S, Ebeid F, el attar Gel D. Impact of drug dosage and brand on bioavailability and efficacy of praziquantel. Pharmacological research. 1995;31(1):53-9. Epub 1995/01/01. PubMed PMID: 7784306.
3. Metwally A, Bennett JL, Botros S, Ebeid F. Effect of cimetidine, bicarbonate and glucose on the bioavailability of different formulations of praziquantel. Arzneimittel-Forschung/Drug Research. 1995;45-1(4):516-8. PubMed PMID: WOS:A1995QU77700014.
4. Bonate PL, Wang TL, Passier P, Bagchus W, Burt H, Lupfert C, et al. Extrapolation of praziquantel pharmacokinetics to a pediatric population: a cautionary tale. Journal of Pharmacokinetics and Pharmacodynamics. 2018;45(5):747-62. doi: 10.1007/s10928-018-9601-1. PubMed PMID: WOS:000445761600008.
5. Masimirembwa CM, Naik YS, Hasler JA. The effect of chloroquine on the pharmacokinetics and metabolism of praziquantel in rats and in humans. Biopharmaceutics & Drug Disposition. 1994;15(1):33-43. doi: 10.1002/bdd.2510150103. PubMed PMID: WOS:A1994MT35100002.
6. Mandour ME, el Turabi H, Homeida MM, el Sadig T, Ali HM, Bennett JL, et al. Pharmacokinetics of praziquantel in healthy volunteers and patients with schistosomiasis. Transactions of the Royal Society of Tropical Medicine and Hygiene. 1990;84(3):389-93. Epub 1990/05/01. PubMed PMID: 2124391.
7. Elguiniady MA, Eltouny MA, Abdelbary MA, Abdelfatah SA, Metwally A. Clinical and pharmacokinetic study of praziquantel in Egyptian schistosomiasis patients with and without liver cell failure. American Journal of Tropical Medicine and Hygiene. 1994;51(6):809-18. doi: 10.4269/ajtmh.1994.51.809. PubMed PMID: WOS:A1994QB81300014.
8. Jung H, Medina R, Castro N, Corona T, Sotelo J. Pharmacokinetic study of praziquantel administered alone and in combination with cimetidine in a single-day therapeutic regimen. Antimicrobial agents and chemotherapy. 1997;41(6):1256-9. doi: 10.1128/aac.41.6.1256. PubMed PMID: WOS:A1997XB71200009.
