# Supplementary material for: Drug metabolism and pharmacokinetics of praziquantel: A review of variable drug exposure during schistosomiasis treatment in human hosts and experimental models
Source: PLoS Negl Trop Dis. 2020 Sep 25;14(9):e0008649. doi: 10.1371/journal.pntd.0008649 (PMC7518612; doi:10.1371/journal.pntd.0008649)
Supplement: S7 Table — HSAC, healthy school age children; PSAC, preschool-aged children; PZQ, praziquantel; SAC, school-aged children. (PDF) [file pntd.0008649.s008.pdf]

S7 Table. Pharmacokinetic parameters of praziquantel (PZQ), PZQ enantiomers, and the major metabolite in Healthy School Age Children (HSAC), infected school-aged children (SAC), and infected preschool-aged children (PSAC) after the administration of racemic PZQ. The PZQ brands, when named, were listed in subscript: BILT: Biltricide. Unless otherwise stated the following acronyms represent; AUC: Area under the plasma concentration-time curve, A ($AUC_{0\text{-}8}$): Area under the plasma concentration-time curve from time zero to time t, B ($AUC_{0\text{-}inf}$): Area under the plasma concentration-time curve extrapolated from time zero to infinity, C (AUC_{last}): Area under the plasma concentration-time curve from time zero to time of last measurable concentration, $t_{1/2}$: Elimination half-life, C_{max}: Peak plasma concentration, T_{max}: Time to reach peak plasma concentration following drug administration.

Drug Administered	Drug Measured	PZQ Dose (mg/kg)	N	$t_{1/2}$ (h)	T_{max} (h)	C_{max} (ng/ml)	AUC	
							(ng/h/ml)	(ng*h/ml)
PZQ [1]		20						
PSAC (haematobium)	*(R)-PZQ*		39	7.39	1.00	320		1160[C]
PSAC (mansoni)			32	5.02	1.50	330		1350[C]
SAC (haematobium)			45	4.41	2.50	180		1010[C]
SAC (mansoni)			47	3.12	1.00	70		270[C]
PSAC (haematobium)	*(S)-PZQ*		39	5.58	1.00	600		1800[C]
PSAC (mansoni)			32	4.32	1.50	500		1820[C]
SAC (haematobium)			45	4.92	1.50	340		1670[C]
SAC (mansoni)			47	3.59	1.00	190		600[C]
PSAC (haematobium)	*(R)-4-OH-PZQ*		39	3.49	2.00	4920		23080[C]
PSAC (mansoni)			32	2.88	2.50	3240		16840[C]
SAC (haematobium)			45	3.28	2.50	4640		22200[C]
SAC (mansoni)			47	2.73	2.00	6430		29870[C]
$PZQ_{(BILT)}$ [2]		30						
HSAC	*PZQ*		1	x	1.50	160	761[A]	
HSAC			1	4.2	1.50	817	2870[A]	
HSAC			1	4.2	1.50	820	2867[A]	
HSAC			1	1.1	2.00	2096	4437[A]	
HSAC			1	1.1	2.00	2365	4753[A]	
HSAC			1	2.8	2.00	1022	2508[A]	
HSAC			6	2.7	1.75	1213	30327[A]	
SAC (haematobium)			1	2.7	1.50	4822	1419[A]	
SAC (haematobium)			1	1.3	1.00	2346	3742[A]	
SAC (haematobium)			1	1.4	1.50	4088	6126[A]	
SAC (haematobium)			1	2	1.50	500	1061[A]	
SAC (haematobium)			1	0.8	4.00	3868	8081[A]	
SAC (haematobium)			5	1.7	1.90	3125	40858[A]	
PZQ [1, 3]		40						
PSAC (haematobium)	*(R)-PZQ*		43	5.57	1.50	410		1170[C]
PSAC (mansoni)			29	5.65	1.00	490		1710[C]

	SAC (haematobium)			46	4.33	2.00	320		1150[C]
	SAC (mansoni)			30	3.48	3.28	131100	500[B]	
	SAC (mansoni)			46	3.91	1.50	250		780[C]
	PSAC (haematobium)	*(S)-PZQ*		43	4.94	1.50	910		2240[C]
	PSAC (mansoni)			29	4.64	1.00	900		2200[C]
	SAC (haematobium)			46	4.61	2.00	740		2730[C]
	SAC (mansoni)			30	2.96	3.30	581300	2620[B]	
	SAC (mansoni)			46	2.25	1.50	770		2190[C]
	PSAC (haematobium)	*(R)-4-OH-PZQ*		43	3.71	3.00	8140		42590[C]
	PSAC (mansoni)			29	3.42	2.00	6920		32740[C]
	SAC (haematobium)			46	3.7	3.00	8160		47790[C]
	SAC (mansoni)			46	3.09	2.50	10320		56590[C]
PZQ [1, 3]			60						
	PSAC (haematobium)	*(R)-PZQ*		41	5.09	1.5	620		2250[C]
	PSAC (mansoni)			33	5.95	1	690		2740[C]
	SAC (haematobium)			44	5.34	2.5	440		1840[C]
	SAC (mansoni)			28	4.36	3.13	144950	430[B]	
	SAC (mansoni)			42	4.49	1.5	290		1000[C]
	PSAC (haematobium)	*(S)-PZQ*		41	4.8	2	1220		4870[C]
	PSAC (mansoni)			33	4.58	1	1330		4990[C]
	SAC (haematobium)			44	5.58	2.5	1250	2580[B]	5090[C]
	SAC (mansoni)			28	3.02	3.23	695560		
	SAC (mansoni)			42	4.05	2	1000		3500[C]
	PSAC (haematobium)	*(R)-4-OH-PZQ*		41	3.64	3	13490		107440[C]
	PSAC (mansoni)			33	3.54	2.5	8290		49940[C]
	SAC (haematobium)			44	3.86	4.5	11490		88320[C]
	SAC (mansoni)			42	3.19	3	13570		89220[C]

References

1. Kovac J, Meister I, Neodo A, Panic G, Coulibaly JT, Falcoz C, et al. Pharmacokinetics of Praziquantel in Schistosoma mansoni- and Schistosoma haematobium-Infected School- and Preschool-Aged Children. Antimicrobial agents and chemotherapy. 2018;62(8). doi: 10.1128/AAC.02253-17;e02253-17. PubMed PMID: WOS:000440008400078.
2. Ofori-Adjei D, Adjepon-Yamoah KK, Lindstrom B. Oral praziquantel kinetics in normal and Schistosoma haematobium-infected subjects. Therapeutic drug monitoring. 1988;10(1):45-9. Epub 1988/01/01. PubMed PMID: 3131934.
3. Bustinduy AL, Waterhouse D, de Sousa-Figueiredo JC, Roberts SA, Atuhaire A, Van Dam GJ, et al. Population Pharmacokinetics and Pharmacodynamics of Praziquantel in Ugandan Children with Intestinal Schistosomiasis: Higher Dosages Are Required for Maximal Efficacy. mBio. 2016;7(4). Epub 2016/08/11. doi: 10.1128/mBio.00227-16. PubMed PMID: 27507822; PubMed Central PMCID: PMCPMC4992966.
