# Supplementary material for: Drug metabolism and pharmacokinetics of praziquantel: A review of variable drug exposure during schistosomiasis treatment in human hosts and experimental models
Source: PLoS Negl Trop Dis. 2020 Sep 25;14(9):e0008649. doi: 10.1371/journal.pntd.0008649 (PMC7518612; doi:10.1371/journal.pntd.0008649)
Supplement: S8 Table — PSAC, preschool-aged children; PZQ, praziquantel; SAC, school-aged children. (PDF) [file pntd.0008649.s009.pdf]

S8 Table. Efficacy parameters of racemic praziquantel (PZQ) treatment on infected preschool-aged children (PSAC) and school-aged children (SAC), and the effect of the different brands of PZQ available. The brands, when named, are listed in subscript; BILT: Biltricide, DIS: Distocide. The superscript descriptions show the additional information of the study; (a): Heavy egg load, (b): Moderate egg load, (c): Light egg load.

Drug Administered	PZQ Dose (mg/kg)	N	Egg Reduction Rate (%)	Cure Rate (%)
PZQ [1-3]	20			
PSAC (haematobium)		39	98.6	88.2
PSAC (mansoni)		32	90.8	65.5
SAC (haematobium)		45	97.5	54.6
SAC (mansoni)		47	84.2	30.4
SAC (haematobium)		11	100	100
$SAC_{(BILT)}$ (a) *(mansoni)*		20	96.4	30.0
$SAC_{(BILT)}$ (b) *(mansoni)*		39	86.2	42.4
$SAC_{(BILT)}$ (c) *(mansoni)*		33	72.7	66.7
$SAC_{(DIS)}$ (a) *(mansoni)*		25	98.0	47.1
$SAC_{(DIS)}$ (b) *(mansoni)*		65	94.0	38.7
$SAC_{(DIS)}$ (c) *(mansoni)*		35	86.1	57.1
PZQ [1-4]	40			
PSAC (haematobium)		43	97.1	77.5
PSAC (mansoni)		29	95.3	74.1
SAC (haematobium)		46	98.8	72.5
SAC (mansoni)		46	98.3	68.9
SAC (haematobium)		13	100	100
SAC (mansoni)		30	82.0	70.0
$SAC_{(BILT)}$ (a) *(mansoni)*		30	96.5	73.3
$SAC_{(BILT)}$ (b) *(mansoni)*		26	85.4	52.0
$SAC_{(BILT)}$ (c) *mansoni)*		24	79.5	66.7
$SAC_{(DIS)}$ (a) *(mansoni)*		30	99.9	90
$SAC_{(DIS)}$ (b) *(mansoni)*		15	93.4	46.7
$SAC_{(DIS)}$ (c) *(mansoni)*		24	90.3	70.9
PZQ [1, 2, 4]	60			
PSAC (haematobium)		41	96.3	67.6
PSAC (mansoni)		33	97.6	78.1
SAC (haematobium)		44	97.5	63.4
SAC (mansoni)		42	99.1	82.9
SAC (haematobium)		11	99.4	90.9
SAC (mansoni)		28	91	82

References

1. Kovac J, Meister I, Neodo A, Panic G, Coulibaly JT, Falcoz C, et al. Pharmacokinetics of Praziquantel in Schistosoma mansoni- and Schistosoma haematobium-Infected School- and Preschool-Aged Children. Antimicrobial agents and chemotherapy. 2018;62(8). doi: 10.1128/AAC.02253-17;e02253-17. PubMed PMID: WOS:000440008400078.
2. Kovac J, Panic G, Neodo A, Meister I, Coulibaly JT, Schulz JD, et al. Evaluation of a novel micro-sampling device, Mitra (TM), in comparison to dried blood spots, for analysis of praziquantel in Schistosoma haematobium-infected children in rural Cote d'Ivoire. Journal of Pharmaceutical and Biomedical Analysis. 2018;151:339-46. doi: 10.1016/j.jpba.2018.01.030. PubMed PMID: WOS:000426025700041.
3. Metwally A, Bennett J, Botros S, Ebeid F, el attar Gel D. Impact of drug dosage and brand on bioavailability and efficacy of praziquantel. Pharmacological research. 1995;31(1):53-9. Epub 1995/01/01. PubMed PMID: 7784306.
4. Bustinduy AL, Waterhouse D, de Sousa-Figueiredo JC, Roberts SA, Atuhaire A, Van Dam GJ, et al. Population Pharmacokinetics and Pharmacodynamics of Praziquantel in Ugandan Children with Intestinal Schistosomiasis: Higher Dosages Are Required for Maximal Efficacy. mBio. 2016;7(4). Epub 2016/08/11. doi: 10.1128/mBio.00227-16. PubMed PMID: 27507822; PubMed Central PMCID: PMCPMC4992966.
