# Supplementary material for: Drug metabolism and pharmacokinetics of praziquantel: A review of variable drug exposure during schistosomiasis treatment in human hosts and experimental models
Source: PLoS Negl Trop Dis. 2020 Sep 25;14(9):e0008649. doi: 10.1371/journal.pntd.0008649 (PMC7518612; doi:10.1371/journal.pntd.0008649)
Supplement: S9 Table — PZQ, praziquantel. (PDF) [file pntd.0008649.s010.pdf]

S9 Table. The comparison of dosing racemic praziquantel (PZQ) alone, or with drug 'B' on PZQ's antischistosomal efficacy in rodent models. The superscript descriptions show the additional information of the study; (e): Without H_2O, (f):Sudanese population, (g): High oil diet, (h): Low oil diet, (n): Egyptian population not cured after 2nd dose PZQ, (k): Egyptian population, (l): Ureamic patient, (m): Egyptian population not cured after dose PZQ, (i): CIM dosed 1h prior to PZQ, (j): CHQ dosed 2h before PZQ. Unless otherwise stated the following acronyms represent; M: Mouse, H: Hamster, WBR: Worm burden reduction, MEF: Mefloquine, ABT: 1-aminobenzotriazole, ART: Artemether, NIL: Nilutamide, MET: Metrifonate, DEX: Dexamethasone, CLO: Clonazepam, TUM: Turmeric.

Drug Administered	Drug Effect Measured	PZQ Dose (mg/kg)	B Dose (mg/kg)	N	WBR (%)
PZQ [1-5]					
M (f) (mansoni)	*PZQ*	50	-	4	13
M (mansoni)				5	16
H (mansoni)		75		5	2
M (f) (mansoni)		100		6	15
M (mansoni)				5	17.8
H (mansoni)				5	36
M (mansoni)	*PZQ*	200		14	56.6
M (mansoni)	*(R)-PZQ*			6	68
M (mansoni)		400		6	97
M (mansoni)				4	84
M (mansoni)	*PZQ*	500		10	95
M (mansoni)		(5 x 12.5)		8	23.07
M (mansoni)		(5 x 25)		8	37.69
M (mansoni)		(5 x 50)		8	65.75
M (mansoni)		(5 x 100)		8	85.75
M (mansoni)		(5 x 200)		8	91.53
PZQ + ABT [1]					
M (g) (mansoni)	*(R)-PZQ*	50	50	2	21
M (h) (mansoni)				6	53
M (g) (mansoni)		100	20	5	60
M (h) (mansoni)			50	5	79
M (g) (mansoni)		200	20	5	100
M (h) (mansoni)			50	4	88
PZQ + ART [5, 6]					
H (i) (mansoni)	*PZQ*	75	150	5	81
H (j) (mansoni)				5	84
H (k) (mansoni)				5	82
H (l) (mansoni)				5	84
H (m) (mansoni)				5	77

H [(i)] *(mansoni)*				5	68
H [(j)] *(mansoni)*				5	76
H [(k)] *(mansoni)*				5	73
H [(l)] *(mansoni)*				5	81
H [(k)] *(mansoni)*		100	300	5	95
H [(l)] *(mansoni)*				5	99
H [(m)] *(mansoni)*				5	92
H [(k)] *(mansoni)*				5	92
H [(l)] *(mansoni)*				5	96
H [(m)] *(mansoni)*				5	90
M (mansoni)		(5 x 12.5)	400	8	56.52
M (mansoni)		(5 x 25)		8	73.07
M (mansoni)		(5 x 50)		8	80.37
M (mansoni)		(5 x 100)		8	85.75
M (mansoni)		(5 x 200)		8	93.44
PZQ + CLO [2]					
M (mansoni)	*PZQ*	200	10	14	61
PZQ + DEX [1]					
M [(n)] *(mansoni)*	*(R)-PZQ*	400	100	4	73
PZQ + MEF [3]					
M [(e)] *(mansoni)*	*PZQ*	42.5	15	4	19.2
M [(f)] *(mansoni)*		50	50	8	25
M [(f)] *(mansoni)*				8	61.5
M [(f)] *(mansoni)*				8	47.8
M [(e)] *(mansoni)*		85	30	4	51.6
M [(f)] *(mansoni)*		100	100	8	86
M [(f)] *(mansoni)*				9	93.1
M [(f)] *(mansoni)*				9	54.7
M [(e)] *(mansoni)*		170	60	4	91.8
M [(e)] *(mansoni)*		340	120	4	97.8
PZQ + MET					
M (mansoni)		(5 x 100)	(5 x 50)	X	95.1
PZQ + NIL [4]					
M (mansoni)	*PZQ*	50	100	5	11.7
M (mansoni)		100	100	5	3.1
M (mansoni)		100	200	5	90.6
PZQ + TUM [7]					
M (mansoni)	*PZQ*	500	400	10	100

References

1. Abla N, Keiser J, Vargas M, Reimers N, Haas H, Spangenberg T. Evaluation of the pharmacokinetic-pharmacodynamic relationship of praziquantel in the schistosoma mansoni mouse model: Possible clinical implications. Tropical Medicine and International Health. 2017;22 (Supplement 1):136-7. doi: http://dx.doi.org/10.1111/%28ISSN%291365-3156. PubMed PMID: 618977725.
2. Araujo N, De Mattos ACA, Coelho PMZ, Katz N. Association of oxamniquine praziquantel and clonazepam in experimental Schistosomiasis mansoni. Memorias do Instituto Oswaldo Cruz. 2008;103(8):781-5. PubMed PMID: 354009130.

3. Keiser J, Manneck T, Vargas M. Interactions of mefloquine with praziquantel in the schistosoma mansoni mouse model and in vitro. Journal of Antimicrobial Chemotherapy. 2011;66(8):1791-7. doi: http://dx.doi.org/10.1093/jac/dkr178. PubMed PMID: 362161771.
4. Keiser J, Vargas M, Vennerstrom JL. Activity of antiandrogens against juvenile and adult Schistosoma mansoni in mice. Journal of Antimicrobial Chemotherapy. 2010;65(9):1991-5. doi: http://dx.doi.org/10.1093/jac/dkq233. PubMed PMID: 359359597.
5. Utzinger J, Chollet J, You JQ, Mei JY, Tanner M, Xiao SH. Effect of combined treatment with praziquantel and artemether on Schistosoma japonicum and Schistosoma mansoni in experimentally infected animals. Acta Tropica. 2001;80(1):9-18. doi: 10.1016/s0001-706x(01)00138-3. PubMed PMID: WOS:000170709200002.
6. Botros SS, Hammam OA, Mahmoud M, Bergquist R. Praziquantel efficacy in mice infected with PZQ non-susceptible S. mansoni isolate treated with artemether: Parasitological, biochemical and immunohistochemical assessment. Apmis. 2010;118(9):692-702. doi: http://dx.doi.org/10.1111/j.1600-0463.2010.02645.x. PubMed PMID: 359413192.
7. Hussein A, Rashed S, El Hayawan I, El-Sayed R, Ali H. Evaluation of the Anti-schistosomal Effects of Turmeric (Curcuma longa) Versus Praziquantel in Schistosoma mansoni Infected Mice. Iranian Journal of Parasitology. 2017;12(4):587-96. PubMed PMID: WOS:000418756700014

.
