# Supplementary material for: Drug metabolism and pharmacokinetics of praziquantel: A review of variable drug exposure during schistosomiasis treatment in human hosts and experimental models
Source: PLoS Negl Trop Dis. 2020 Sep 25;14(9):e0008649. doi: 10.1371/journal.pntd.0008649 (PMC7518612; doi:10.1371/journal.pntd.0008649)
Supplement: S10 Table — DDI, drug–drug interactions; HNV, healthy normal volunteers; PZQ, praziquantel. (PDF) [file pntd.0008649.s011.pdf]

S10 Table. The pharmacokinetic parameters of praziquantel (PZQ), its enantiomers, and the major metabolite in Healthy Normal Volunteers (HNV) after the administration of racemic PZQ with drug 'B' to evaluate potential drug-drug interactions (DDIs). The brands, when named, were listed in subscript; BILT: Biltricide, DIS: Distocide. The superscript descriptions show the additional information of the study: (a): Egyptian Population, (b): CHQ dosed 2h before PZQ, (c): CIM dosed 1h prior to PZQ, (d): Without H_2O. Unless otherwise stated the following acronyms represent; AUC: Area under the plasma concentration-time curve, B ($AUC_{0\text{-}inf}$): Area under the plasma concentration-time curve extrapolated from time zero to infinity, $t_{1/2}$: Elimination half-life, C_{max}: Peak plasma concentration, T_{max}: Time to reach peak plasma concentration following drug administration.

Drug Administered (*Model*)	Drug Measured	PZQ Dose (mg/kg)	B Dose (mg/kg)	N	$t_{1/2}$ (h)	T_{max} (h)	C_{max} (ng/ml)	AUC (ng/ml/h)
PZQ [1]	*PZQ*	20						
HNV(BILT) *(d)*				10	0.97	1.65	846	1303
HNV(DIS) *(d)*				10	1.24	2.60	558	2133
PZQ + BIC [2]	*PZQ*	20	2(g)					
HNV (a)				10	2.15	1.85	563	951
HNV(DIS) *(a)*				10	4.11	1.20	384	956
PZQ + CIM [2, 3]	*PZQ*	20	800					
HNV (a)				10	1.50	1.70	770	2190
HNV(DIS) *(a)*				10	2.66	1.65	835	2428
HNV (c)		(3x25)	400	1	1.80			15770
HNV (c)				1	2.40			21690
HNV (c)				1	1.80			15770
HNV (c)				1	2.30			8340
HNV (c)				1	2.40			8950
HNV (c)				1	1.90			10370
HNV (c)				1	2.20			16180
HNV (c)				1	2.40			16580
PZQ + GLUC [2]	*PZQ*	20	50(g)					
HNV (a)				10	2.70	2.10	373	1130
HNV(DIS) *(a)*				10	2.46	2.11	449	1094
PZQ + KTZ [4]	*PZQ*	20	500(mg)					
HNV				29	5.16	2.13	2266	6172 (ng*h/mL)[B]
PZQ (BILT) + CHQ [5]	*PZQ*	40	600					
HNV (b)				1	1.41	3.00	430	1620
HNV (b)				1	1.54	1.75	2100	9420
HNV (b)				1	0.99	2.00	330	1420

HNV [b]				1	2.57	1.50	400	2200
HNV [b]				1	2.82	2.00	1890	10080
HNV [b]				1	1.39	1.25	78	360
HNV [b]				1	2.57	2.00	4380	35920
HNV [b]				1	1.90	2.00	950	4060
HNV [b]				8	1.81	1.93	880	4170
PZQ + ABZ [6]		1500	400					
HNV	*(S)-PZQ*			9	1.67	2.76	450	3030[B]
HNV	*(R)-PZQ*			9	1.49	2.73	240	1530[B]
HNV	*(S)-4-OH-PZQ*			9	2.14	3.27	1070	9120[B]
HNV	*(R)-4-OH-PZQ*			9	1.84	2.77	790	5380[B]

References

1. Metwally A, Bennett J, Botros S, Ebeid F, el attar Gel D. Impact of drug dosage and brand on bioavailability and efficacy of praziquantel. Pharmacological research. 1995;31(1):53-9. Epub 1995/01/01. PubMed PMID: 7784306.
2. Metwally A, Bennett JL, Botros S, Ebeid F. Effect of cimetidine, bicarbonate and glucose on the bioavailability of different formulations of praziquantel. Arzneimittel-Forschung/Drug Research. 1995;45-1(4):516-8. PubMed PMID: WOS:A1995QU77700014.
3. Jung H, Medina R, Castro N, Corona T, Sotelo J. Pharmacokinetic study of praziquantel administered alone and in combination with cimetidine in a single-day therapeutic regimen. Antimicrobial agents and chemotherapy. 1997;41(6):1256-9. doi: 10.1128/aac.41.6.1256. PubMed PMID: WOS:A1997XB71200009.
4. Nleya L, Thelingwani R, Li XQ, Cavallin E, Isin E, Nhachi C, et al. The effect of ketoconazole on praziquantel pharmacokinetics and the role of CYP3A4 in the formation of X-OH-praziquantel and not 4-OH-praziquantel. European journal of clinical pharmacology. 2019;75(8):1077-87. doi: 10.1007/s00228-019-02663-8. PubMed PMID: WOS:000475794400006.
5. Masimirembwa CM, Naik YS, Hasler JA. The effect of chloroquine on the pharmacokinetics and metabolism of praziquantel in rats and in humans. Biopharmaceutics & Drug Disposition. 1994;15(1):33-43. doi: 10.1002/bdd.2510150103. PubMed PMID: WOS:A1994MT35100002.
6. Lima RM, Ferreira MAD, Carvalho T, Fernandes BJD, Takayanagui OM, Garcia HH, et al. Albendazole-praziquantel interaction in healthy volunteers: kinetic disposition, metabolism and enantioselectivity. British journal of clinical pharmacology. 2011;71(4):528-35. doi: 10.1111/j.1365-2125.2010.03874.x. PubMed PMID: WOS:000288249500008.
