# Supplementary material for: Drug metabolism and pharmacokinetics of praziquantel: A review of variable drug exposure during schistosomiasis treatment in human hosts and experimental models
Source: PLoS Negl Trop Dis. 2020 Sep 25;14(9):e0008649. doi: 10.1371/journal.pntd.0008649 (PMC7518612; doi:10.1371/journal.pntd.0008649)
Supplement: S11 Table — PZQ, praziquantel. (PDF) [file pntd.0008649.s012.pdf]

S11 Table. The comparison of racemic praziquantel (PZQ) alone, and with drug 'B' in reducing hepatic granuloma diameter in murine models. The brands, when named, were listed in subscript; DIS: Distocide. The superscript descriptions show the additional information of the study; (a): PZQ-susceptible *S. mansoni* isolate CD, (b): PZQ-non-susceptible *S. mansoni* isolate EE10. Unless otherwise stated the following acronyms represent; M: Mouse, MEF: Mefloquine, PTX: Pentoxifylline, AdDP: Adamantylamide dipeptide, TUM: Turmeric.

Drug Administered	Drug Effect Measured	PZQ Dose (mg/kg)	B Dose (mg/kg)	N	Reduction in Hepatic Granuloma Diameter (%)
PZQ [1-4]					
M (a) *(mansoni)*	*PZQ*	(3 x 100)	-	9	18.86
M (b) *(mansoni)*				9	13.61
M (a) *(mansoni)*		(2 x 500)		9	20.58
M (b) *(mansoni)*				9	17.32
M (mansoni)		500		10	46.4
M (DIS) *(mansoni)*		(2 x 100)		9	15.50
M (DIS) *(mansoni)*		200		8	28.08
M (DIS) *(mansoni)*		(2 x 500)		9	49.50
M (DIS) *(mansoni)*		1000		8	39.39
PZQ + AdDP [1]					
CDM (mansoni)		(3 x 100)	5	10	20.95
EE10M (mansoni)				10	18.47
PZQ (DIS) + MEF [3]					
M (mansoni)		200	200	8	43.23
M (mansoni)		1000	400	8	44.97
PZQ (DIS) + PTX [2]					
M (mansoni)		(2 x 100)	(5 x 10)	9	5.40
M (mansoni)				9	27.00
PZQ + TUM [4]					
M (mansoni)		500	400	10	28.60

References

1. Botros S, Mahmoud M, Hammam O, Salah F, Zidek Z, Masek K. Effect of adamantylamide dipeptide as adjuvant therapy to praziquantel in mice infected with different S. mansoni isolates. Apmis. 2006;114(7-8):480-91. doi: http://dx.doi.org/10.1111/j.1600-0463.2006.apm_380.x. PubMed PMID: 44252502.

2. El-Lakkany N, Nosseir M. Pharmacodynamics of pentoxifylline and/or praziquantel in murine schistosomiasis mansoni. Apmis. 2007;115(3):184-94. doi: 10.1111/j.1600-0463.2007.apm_501.x. PubMed PMID: WOS:000244881000002.
3. El-Lakkany NM, el-Din SHS, Sabra A, Hammam OA. Pharmacodynamics of mefloquine and praziquantel combination therapy in mice harbouring juvenile and adult Schistosoma mansoni. Memorias Do Instituto Oswaldo Cruz. 2011;106(7):814-22. doi: 10.1590/s0074-02762011000700006. PubMed PMID: WOS:000297789800006.
4. Hussein A, Rashed S, El Hayawan I, El-Sayed R, Ali H. Evaluation of the Anti-schistosomal Effects of Turmeric (Curcuma longa) Versus Praziquantel in Schistosoma mansoni Infected Mice. Iranian Journal of Parasitology. 2017;12(4):587-96. PubMed PMID: WOS:000418756700014.
