# Supplementary material for: Drug metabolism and pharmacokinetics of praziquantel: A review of variable drug exposure during schistosomiasis treatment in human hosts and experimental models
Source: PLoS Negl Trop Dis. 2020 Sep 25;14(9):e0008649. doi: 10.1371/journal.pntd.0008649 (PMC7518612; doi:10.1371/journal.pntd.0008649)
Supplement: S12 Table — (PDF) [file pntd.0008649.s013.pdf]

S12 Table. Comparison of the results of this systematic review in comparison to tuberculosis. The same search criteria was applied to the drugs used to treat tuberculosis.

	**Schistosomiasis**	**Tuberculosis**
Search Criteria	*(praziquantel OR PZQ) AND (pharmaco*) AND (schistosom* OR bilharzia)*	*TS=(Isoniazid OR Rifampin OR Rifadin OR Rimactane OR Ethambutol OR Myambutol OR Pyrazinamide) AND TS=(pharmaco*) AND TS=(tuberculosis OR TB)*
PubMed	87 Results	1210 Results
Web of Science	152 Results	1181 Results
EMBASE	167 Results	1276 Results
